# Nano-spanlastics-loaded dissolving microneedle patches for ketotifen fumarate: advanced strategies for allergic conjunctivitis treatment and molecular insights

**DOI:** 10.1007/s13346-025-01796-x

**Published:** 2025-02-11

**Authors:** Sammar Fathy Elhabal, Mohamed El-Nabarawi, Mohamed Fathi Mohamed Elrefai, Mahmoud H. Teaima, Mai S. Shoela, Gehad M. Khamis, Ahmed Mohsen Faheem, Nada ahmed kholeif, Mahmoud Tarek Sanad

**Affiliations:** 1 Department of Pharmaceutics and Industrial Pharmacy, Faculty of Pharmacy, Modern University for Technology and Information (MTI), Mokattam, Cairo, 11571 Egypt; 2 Department of Pharmaceutics and Industrial Pharmacy, Faculty of Pharmacy, Cairo University, Cairo, 11562 Egypt; 3 Department of Anatomy, Histology, Physiology and Biochemistry, Faculty of Medicine, The Hashemite University, Zarqa, 13133 Jordan; 4 Department of Anatomy and Embryology, Faculty of Medicine, Ain Shams University, Cairo, 11566 Egypt; 5 Department of Clinical Pharmacology, Faculty of Medicine, Alexandria University, Alexandria, 21526 Egypt; 6 Department of Medical Biochemistry and Molecular Biology, Faculty of Medicine, Mansoura University, Mansoura, Egypt; 7 Department of Pathology, Faculty of Medicine, Cairo University, Cairo, Egypt; 8 Department of Pharmaceutics and Industrial Pharmacy, Faculty of Pharmacy, Galala University, New Galala, 43713 Egypt

**Keywords:** Allergic conjunctivitis, Ketotifen Fumarate, Spanlastics, Ovalbumin, Polymerase Chain Reaction (PCR)

## Abstract

Allergic conjunctivitis (AC) is the most common inflammatory disease affecting the eye's ocular surface, lid, conjunctiva, and cornea. However, effective ocular drug delivery remains challenging due to physiological barriers such as the corneal barrier. Ketotifen (KF), a widely used antihistamine and mast cell stabilizer, for treating AC and atopic asthma but belongs to the Biopharmaceutical Classification System (BCS II) have poor solubility. This study developed a multiple strategies approach for the first time, utilizing the spanlastics nano-vesicular carriers’ system (SP) containing KF using an ethanol injection method. The optimized KF-SP exhibited the smallest particle size, largest zeta-potential and entrapment efficiency ∼232.5 ± 1.9 nm, -28 ± 0.51 and 73 ± 0.02%, respectively were further incorporated into PVA/PVP polymeric dissolving microneedles (MNs) by using a micromolding technique. Scanning electron microscopy (SEM) analysis confirmed well-defined tips and morphology, and in vitro studies showed a controlled 93% cumulative release over 72 h, with a zero-order kinetic release profile, providing stable therapeutic levels. Pharmacodynamic evaluation using the Ovalbumin/Aluminium hydroxide-induced AC model demonstrated significant reductions in IgE, TNF-α, and IL-6 levels by 68.7%, 71.3%, and 67.6%, respectively, while TGF-β and IL-10 levels increased by 70.1% and 62.7% using ELISA (Enzyme-Linked Immunosorbent Assay). Gene expression analysis (IGF-1, Annexin A1, and Bcl2) further supported the therapeutic potential of this system. In this study, we proved the topical application of the multiple strategies approach KF-SP loaded PVA/PVP MNs patch offers a targeted, sustained release treatment for AC, with promising implications for prolonged ocular therapy.

**Graphical Abstract:**

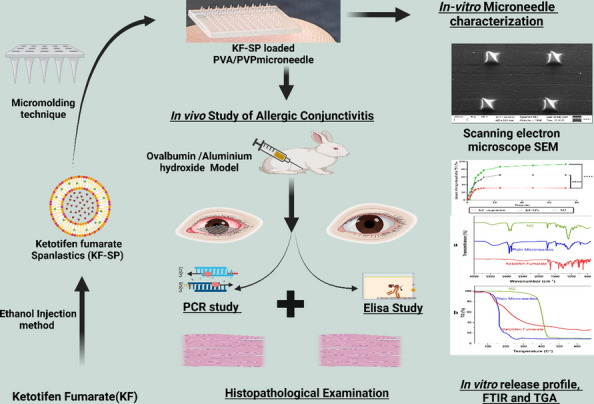

**Supplementary Information:**

The online version contains supplementary material available at 10.1007/s13346-025-01796-x.

## Introduction

Allergic conjunctivitis (AC)is an inflammatory disease affecting the eye's ocular surface, lid, conjunctiva, and cornea. AC is an abnormal immune hypersensitivity, an immune-mediated ocular condition that can take many forms, including seasonal allergic conjunctivitis (SAC) and perennial allergic conjunctivitis (PAC) [1]. AC is an inflammatory condition impacting the ocular surface, eyelids, conjunctiva, and cornea. AC is an aberrant immunological hypersensitivity, an immune-mediated ocular disorder that manifests in various forms, including seasonal allergic conjunctivitis (SAC) and perennial allergic conjunctivitis (PAC) [2]. The presence of pollen from grass, weeds, and trees is linked to the symptoms experienced by SAC sufferers. Individuals diagnosed with PAC display a persistent sensitivity to typical household allergens, including molds, animal dander, and dust mites. Allergic reactions can be broadly classified into type I and type IV, with type I reactions being the primary cause of AC [3]. Itchy eyes are the most prevalent symptom of AC, with most patients additionally experiencing photophobia, burning sensations, increased secretions, and lacrimation. A corneal infection may jeopardize the vision of some individuals. Individuals are more susceptible to allergens because of the frequent application of contact lenses and ocular cosmetics, heightened irritation from air pollution, and more causes [4]. Exported data from outside of China indicates that immunoglobulin E (IgE)-mediated allergy responses affect over 25% of the population [5]. In Western medicine, antihistamines, short-term corticosteroids, mast cell stabilizers, and sublingual or subcutaneous immunotherapy are the most used approaches. Antibodies such as cyclosporine eye drops are used as immunosuppressants for patients with severe AC [3]. The several pharmaceutical classes that currently comprise topical antiallergy medications include antihistamines, nonsteroidal anti-inflammatory medicines, mast-cell stabilizers, dual-acting compounds (mast-cell stabilizers with antihistamine action), and others. calcineurin inhibitors, corticosteroids, and vasoconstrictors always cause excruciating and occasionally incapacitating symptoms, including intense tearing, chemosis, erythema, and ocular oedema [6].

Ketotifen (KF) is an antihistamine medicine that acts by inhibiting the production of histamine, a substance in the body that triggers allergic reactions. It is used to treat AC and moderate atopic asthma as a mast cell stabilizer and histamine H1 receptor blocker [7]. It is an asthma medicine that can successfully lessen the frequency, intensity, and length of asthma symptoms or attacks in children when used regularly in conjunction with other anti-asthmatic drugs. Its use may potentially assist minimize the requirement for extra daily anti-asthmatic drugs [8]. Therefore, it is crucial to accurately identify KF in biological fluids and medications. KF belongs to the Biopharmaceutical Classification System (BCS II) have low solubility and high permeability, One of the main drawbacks of KF, low ocular bioavailability and low patient compliance [9].

Spanlastics (SP), or modified niosomes, are span-based elastic vesicles. Like niosomes, SP are spherical, unilamellar structures that are uni-or multilamellar and mostly made of lipophilic non-ionic surfactant (span) with an extra edge activator to giving their walls flexibility, by reducing the surface tension of the vesicles, edge activators (single chain surfactants) can destabilize them and increase their vesicular bilayer deformability, so SP high flexibility made it easier for drugs to pass through different mucosal biomembranes, like skin, cornea, gastrointestinal mucosa, and more, by going through membrane holes with little chance of vesicular rupture [9, 10]. The potential of SP to facilitate medication absorption through the skin, intestines, and eyes has been investigated previously. SP's polymer matrix and surfactants work together to encase the medicine, making it more soluble and permeable [11].

Dissolving microneedles (DMNs) have garnered increasing interest due to their potential for superior drug loading, solubility and permeability [12]. When the needles come into touch with the ocular, aqueous mixes of polymers can penetrate the outer layers of the eye effectively without causing damage or significant discomfort. Poly (vinyl pyrrolidone) (PVP), and polyvinyl alcohol (PVA) are polymers, PVP was most frequently utilized in drug-containing DMNs, with hyaluronic acid and PVA coming in a close second, these polymers are biocompatible, non-toxic, and have received approval from the FDA and the European Medicines Agency (EMA) [13].

This work aimed to formulate and evaluate KF in SP using span and different edge activator (EA) percentages. Furthermore, adopting Box-Behnken statistical design to investigate the main influences and interactions of independent variables on the physicochemical properties of the prepared (Ketotifen fumarate loaded spanlastics) KF-SP such as particle size, entrapment efficiency % (EE %), polydispersity index (PDI), zeta potential (ZP) and, in vitro release characteristics. KF-SP loaded with PVA/PVP dissolvable microneedle prepared by a layered casting approach using a dissolvable polymer composite. To improve the deliverability and comparability with commercially available dosage forms. To address the difficulties of efficient ocular drug delivery, numerous methods have been created. For example, hydrogels offer continuous medication release and are biocompatible. However, their reliance on passive diffusion and limited drug-loading capacity frequently lead to less-than-ideal therapeutic results, particularly for disorders that call for precise and localized drug concentrations. Similarly, contact lens-based solutions provide longer-lasting drug release while in use, but they also have drawbacks such as fluctuating tear film dynamics, patient noncompliance, and possible discomfort from lengthy usage. Another major challenge is maintaining constant drug release kinetics from contact lenses [14]. The KF-SP loaded Microneedle system, on the other hand, offers a unique technique that specifically tackles these constraints. This method bypasses natural obstacles including tear flow and corneal epithelial resistance by administering medications using microneedles that pierce the ocular surface, resulting in accurate and reliable drug deposition at the intended location. Its dissolvable nature also guarantees simplicity and safety by doing away with the necessity for patient compliance with external devices or retrieval. For the treatment of ocular disorders like conjunctivitis, these benefits make the KF-SP-loaded MN system a viable substitute for conventional hydrogels and contact lens-based platforms. The objective of this study was to evaluate the newly designed KF-SP loaded PVA/PVP MNs for their effectiveness in ophthalmic delivery of KF in-vivo noninfectious AC in a rabbit model, Gene expression levels of IGF1, Annexin A1, and Bcl2 were quantified using qRT-PCR, and ELISA (Enzyme-Linked Immunosorbent Assay) for growth factor-β (TGF-β) immunoglobulin E (IgE), interleukin-10 (IL-10), tumour necrosis factor-α (TNF-α), and interleukin-6 (IL-6) markers.

## Materials and methods

### Materials

Ketotifen fumarate (KF) was provided by Sigma Chemical Co., St Louis, Mo, USA. Polyoxyethylene sorbitan monooleate (Tween 80), Sorbitan monostearate (Span 60), and ethanol (99.9%) were purchased from Piochem (6th of October, Egypt). Kolliphor RH 40 was purchased from BASF (Ludwigshafen, Germany). Brij 35, Polyvinyl pyrrolidone K-30 (PVP K-30, MW 40,000 Da), (PVA; MW 160,000 Da), Ovalbumin (OVA) and aluminium hydroxide (AH) were derived from Sigma-Aldrich Chemical Co. (St. Louis, MO, USA). Monosodium phosphate, disodium phosphate and sodium chloride were acquired from El-Nasr Pharmaceutical Chemicals Co. (Cairo, Egypt). Spectra Por© semi-permeable membrane tubing (12,000–14,000 MWCO) was obtained from Spectrum Laboratories Inc. (CA, USA). Any other chemicals used were highly analytical and were used as requested.

## HPLC method of ketotifen fumarate

HPLC analysis was carried out using an Agilent 1260 series (Agilent Technologies, USA) for KF samples analysis. the column used was Agilent C18 (4.6 mm × 250 mm i.d., 5 μm) (Phenomenex, USA) was used to separate the sample components. The mobile phase composition included acetonitrile: phosphate buffer PH = 4 (0.1 M Na_2_HPO_4_ PH determined by glacial acetic acid) (30: 70) ratio, respectively. A sample (20 μl) was injected, and the chromatography was performed at a flow rate of 1.2 mL/min. Detection was carried out at 300 nm using a diode array detector (DAD), with the column temperature set at 25 °C. The concentration of KF was determined using a standard calibration curve obtained between a concentration range of 2.5 to 40 μg/ml with a correlation coefficient of 0.9999.

## Formulation of KF-loaded spanlastics

The ethanol injection method employed by Kumar was utilized to prepare KF-loaded SP (KF-SP) [15]. Specifically, KF and Span 60 were dissolved in ethanol in a 60 °C water bath. A preheated aqueous phase which had a dissolved edge activator (EA) (Tween 80, Kolliphor Rh 40, or Brij 35), was prepared. The KF solution was injected into it slowly with a fixed 1:5 ratio organic phase: aqueous phase for Different Span 60 to EA ratios which were prepared with the same previous method. The solution was stirred on a magnetic stirrer constantly at a speed of 800 (rpm) at the specified temperature, allowing all the ethanol to evaporate completely, resulting in the formation of an aqueous dispersion of SP, following this, ultrasonic water-bath sonication was employed for five minutes to achieve an optimal nanoparticle size within a sonicator water bath (Crest Ultrasonics Corp., NJ, USA) operating at a temperature of 25 °C [9]. The formulated SP are stored for 24 h at 4 °C to provide maturity. The analyzed Span60 to edge activator ratios comprised 80:20, 70:30, and 60:40% W/W, correspondingly. The composition of the examined formulations is detailed in Tables 1 and 2.

**Table 1:** Box-Behnken statistical design for optimization of Ketotifen fumarate-spanlastics

Factors (Independent variables)	Levels		
X_1_: Span 60 /edge activator	60:40	70:20	80:20
Type of edge activator	Kolliphore RH40	Tween 80	Brij
X3: Stirrer time (min)	5	10	15
Responses (Dependent variables)	**Constraints**		
Y_1_: EE (%)	Maximize		
Y_2_: PS (nm)	Minimize		
Y_3_: PDI	Minimize		
Y_4_: ZP	Maximize (absolute value)		

EE%; entrapment efficiency percent, EA; Edge activator; PS; particle size, PDI; polydispersity index, and ZP; zeta potential

**Table 2:** Experimental runs, independent variables, and measured response of the 3^3^ full factorial experimental designs of Ketotifen fumarate-spanlastics

Run	X1 Span 60: EA ratio (%)	X2 Stirrer Time (min)	X3 Type of EA	PS (nm)	PDI (nm)	ZP (mV)	EE (%)
1	80:20	10	Brij	249.65 ± 0.43	0.32 ± 0.05	−26 ± 0.03	37 ± 0.98
2	80:20	10	Kolliphore RH40	315.15 ± 0.23	0.31 ± 0.11	−19 ± 0.34	43 ± 0.05
3	60:40	15	Kolliphore RH40	288.43 ± 0.35	0.45 ± 0.03	−18 ± 0.74	41 ± 0.82
4	70:30	15	Tween 80	227.55 ± 0.52	0.29 ± 0.01	−19 ± 0.65	65 ± 0.34
5	80:20	15	Tween 80	265.25 ± 0.55	0.32 ± 0.01	−27 ± 0.34	72 ± 0.74
6	60:40	10	Tween 80	259.05 ± 0.34	0.26 ± 0.03	−26 ± 0.67	40 ± 0.32
7	70:30	5	Tween 80	342 ± 0.52	0.31 ± 0.04	−21 ± 0.83	65 ± 0.58
8	60:40	5	Brij	271.21 ± 0.63	0.37 ± 0.05	−15 ± 0.86	39 ± 0.63
9	80:20	5	Tween 80	234 ± 0.59	0.54 ± 0.12	−27 ± 0.09	66 ± 0.51
10	60:40	5	Kolliphore RH40	501.12 ± 0.49	0.47 ± 0.14	−17 ± 0.56	55 ± 0.67
11	80:20	10	Tween 80	213 ± 0.45	0.33 ± 0.013	−27 ± 0.69	70 ± 0.41
12	60:40	15	Brij	245.65 ± 0.48	0.32 ± 0.16	−18 ± 0.78	43 ± 0.91
13	70:30	10	Brij	197.55 ± 0.45	0.45 ± 0.02	−18 ± 0.66	40 ± 0.84
O.F	75:25	15	Tween 80	232.5 ± 1.9 nm	0.29 ± 0.01	−28 ± 0.51	73 ± 0.02

EE%; entrapment efficiency percent, EA; Edge activator, O.F; Optimize formula; PS; particle size, PDI; polydispersity index, and ZP; zeta potential

## In vitro characterization of KF-loaded spanlastics

### Dynamic light scattering (DLS) characterization for vesicle size, zeta potential, and polydispersity index

The dynamic light scattering (DLS) technology, implemented via a Zetasizer Nano ZS (Malvern Instruments; Worcestershire, UK), was utilized to assess the hydrodynamic vesicle diameter (z-average), the particle size is determined in three independent batches, with each batch measured in triplicate to ensure batch-to-batch consistency and the polydispersity index (PDI) of the SP. This method involves examining fluctuations in light scattering caused by the Brownian motion of vesicles, enabling the size (z-average) estimation [16, 17]. The ZP was determined by measuring the [17] charged vesicles' electrophoretic mobility with the same apparatus. Every measurement was made at 25 °C. in triplicate following the formulations' dilution [18].

### Evaluation of drug entrapment efficiency (EE) percentages

The quantification of KF encapsulation within SP was achieved through an indirect method, wherein the discrepancy between the initial KF quantity introduced into the vesicles and the residual amount post-separation of the supernatant from the prepared SP was calculated. The separation was achieved by using a cooling ultracentrifugation system (Heraeus Megafuge 1.0 R; Hanau, Germany) set at 4 °C for 2 h at 15,000 rpm [19, 20]. After the appropriate dilution, the unentrapped KF was quantified at a wavelength of 300 nm using a spectrophotometer (Shimadzu UV-1601 PC; Kyoto, Japan). The entrapment efficiency (EE) percentages were subsequently determined according to Eq. (1).

1 $$EE\%=Total\;amount\;of\;Ketotifen-unentrapped\;KF/total\;amount\;of\;KF\;x\;100$$

### Statistical analysis

Using the software of Design-Expert (Version 7, Stat-Ease Inc., USA), a Box-Behnken statistical design was used. The design is summed up in Table 1 showed two factors, X1 (Span 60: EA ratio (w/w)) and X2 (EA Type), each of which had three levels. Following the examination of experimental data and desirability calculation, the best formulation was selected.

## In vitro characterization of optimized formula spanlastics

### Morphological assessment utilizing transmission electron microscopy (TEM)

The morphological assessment of the systems was conducted to investigate structural characteristics including lamellarity, size, and shape uniformity, and to examine the [21] occurrence of aggregated KF-loaded SP. In summary, a drop of appropriately diluted dispersion was applied onto a 300-mesh carbon-coated copper grid and settled for 3 to 5 min. The filter paper was used to remove any excess fluid, and the grid was left to air dry at room temperature for 10 min before examination with a transmission electron microscope (Jeol JEM 1230, Tokyo, Japan) operating at 80 kV [22].

### Fourier transform infrared spectroscopy (FT-IR) analysis

The FT-IR spectral study sought to verify probable physicochemical compatibility and chemical intermolecular interactions among Ketotifen, Span 60, Tween 80, and Ketotifen-loaded spanlastics. Spectral scanning was conducted utilizing an FTIR spectrophotometer (Shimadzu; Kyoto, Japan) across the wavelength range of 4000 to 400 cm^−1^. Spectral smoothing and baseline correction methods were executed. Dry nitrogen gas was employed to cleanse the detector, reducing moisture and augmenting signal intensity [16, 20].

### Differential scanning calorimetry (DSC) analysis

The analysis of differential scanning calorimetry (DSC) assessed the crystallinity levels and probable interactions among Ketotifen, span 60, Tween 80, and KF-loaded SP to evaluate their thermotropic qualities. Samples weighing 3 to 4 mg were subjected to heating in tightly sealed flat-bottomed aluminium pans across a temperature range between 25 to 400 °C while maintaining a heating rate of 10 °C per minute and an inert nitrogen flow of 30 ml per minute. The differential scanning calorimeter (Shimadzu DSC 50; Kyoto, Japan) was calibrated (purified indium (99.9%) and used to record DSC thermograms [23, 24]*.*

### X-ray diffraction (XRD) analysis

X-ray diffraction (XRD) analysis was conducted on KF pure powder, span 60, Tween 80, and lyophilized KF-loaded SP using a Scintag X-ray diffractometer (USA). Cu-radiation with a nickel filter was employed, operating at 40 kV and 40 mA, with a scanning rate of 4 °C/min over a 2θ range spanning from 5° to 80° [21, 25].

### Freeze-drying of ketotifen-loaded Spanlastics solutions

The SP dispersions were loaded with KF and underwent freezing and lyophilization using the Novalyphe-NL 500 lyophilizer (Savant Instruments; NY, USA) for 24 h at 45 °C under a pressure of 7 m bar. Subsequently, solid-state investigations were conducted on the resulting lyophilized SP, as well as on their components and the physical mixture [26].

### Stability study

The effects of three months on colour, PS, PDI, ZP, and EE% were evaluated in a short-term stability study. The characterisation results of three optimized SP formulations determined which ones to explore. The chosen recipes were maintained at 4^O^C and 25^O^C during the investigation [16, 27].

## Fabrication of PVA/PVP-MNs loading freeze-dried KF-loaded SPN

Based on the design criteria established from previous studies, a polydimethylsiloxane (PDMS) mold was fabricated with a 5 × 5 array configuration. The mold design included tip parameters of 500 µm height (H500), 250 µm base width (B250), and 500 µm pitch (P500), which were optimized to achieve uniform microneedle formation. For a single eye, the recommended effective dosage of freeze-dried KF-loaded SPN is (5, 10,15% W/V) [28, 29]. It was dissolved in PVA with different ratios, and PVP as shown in Table 3, after being dissolved in deionised water [12, 30]. Subsequently, the mixture was applied onto the PDMS mold and vacuum treated for 10 min. The mold containing the solution was allowed to dry for 24 h at room temperature in a desiccator that contained silica gel. Eventually, the PDMS molds were carefully peeled to release the PVA/PVP-MNs loading freeze-dried KF-loaded SP, which were then preserved in a desiccator for subsequent usage [31].

**Table 3:** Fabrication of PVA/PVP-MNs loading freeze-dried Ketotifen fumarate-spanlastics

Formulation	Ketotifen fumarate	PVA	PVP
M1	5	15	20
M2	10	10	25
M3	15	5	30

PVA: Polyvinyl Alcohol, and PVP: Polyvinylpyrrolidone and, MNs: Microneedle

## Physical characteristics of the microneedles

### Studies on morphology

The tip diameter, base diameter, and length of seven arrays of KF-SP-PVA/PVP MNs were measured under a microscope. The aspect ratio can be found by dividing the length by the base diameter ratio. Every calculation was done in triplicates, and the mean ± standard deviation was computed [32].

### Studies on drug content

Using a stirring speed of 300 rpm, distilled water containing 2.5% Tween 80 was used to dissolve an array of KF-SP-loaded PVA/PVP MNs magnetic agitators for one hour. The resulting solution was diluted with methanol and sonicated for five minutes to allow the KF from the fabricated KF-SP-loaded PVA/PVP MNs to completely dissolve using the previously validated HPLC method outlined in previous section, the drug content of the produced MNs was ascertained [33].

### Mechanical characterization

Weights 250 g, 500 g, and 1000 g were applied to the tips of the KF-SP-loaded PVA/PVP MNs patch for five minutes before being taken off to measure the DMN's height reduction. The form and fracture of the optical microscope were then used to assess the DMN, which shows the mechanical strength [34].

## Characterization of optimized Microneedle

### Scanning electron microscopy (SEM)

The morphology of the KF-SP-loaded PVA/PVP MNs patch was investigated using a scanning electron microscope (SU8010, HITACHI, Japan). The kF patch was affixed to a microscope carrier, and visualization was conducted at 6 kV with a magnification of 150 × [21].

### Fourier transform infrared (FTIR) analysis

An FTIR spectrometer was used to record the room temperature FTIR spectra of a KF-SP loaded PVA/PVP MNs patch and its constituent parts between 500 and 4000 cm.^−1^ (Tensor II, Germany, Bruker) [35]

### Thermogravimetric analysis (TGA)

Thermogravimetric analysis was used to verify weight loss in response to temperature increases. At a rate of 10 °C per minute, the samples, which included KF, Microneedle without KF, and KF-SP-loaded PVA/PVP MNs patch were heated from 25 °C to 350 °C. The weight loss was quantified to track any modifications in the MN's thermal capacity [22]*.*

## In vitro drug release study

We carried out an in vitro drug release investigation in phosphate-buffered saline to investigate the in vitro drug release from the KF, KF-SP, and KF-SP-loaded PVA-PVP patch. (PBS, pH = 7.4) with 37 °C and 5% Tween 20, MNs patches were, in short, soaked in 2 mL of PBS solution containing 5% Tween 20 and placed in an air bath (37 ◦C). VU spectroscopy was used to quantify the amount of freshly generated release KF that was released, and 1 mL aliquots of release medium were collected for drug measurement at specific intervals [16].

## In vivo study

### Ethical approval

New Zealand Male albino rabbits, averaging weight (2.5 ± 0.5 kg) were obtained from the Cairo University animal sanctuary in Egypt, with 30 mature males divided into five groups. After acclimatization for a week under consistent light–dark cycles, the rabbits were housed in cages with controlled temperature (22–25 °C), and humidity (45–60%). Ethical approval was obtained from the Cairo University, Faculty of Pharmacy Animal Ethics Committee (Approval no. PI 3173). The formulations were sterilized with gamma-irradiation using a C_o_ irradiator at the National Centre for Radiation Research & Technology, a subsidiary of the Atomic Energy Authority in Nasr City, Egypt, sterilized samples in type I glass vials.

### Induction of ocular conjunctivitis

Five sets of rabbits (n = 6) were utilized to test the anti-inflammatory properties of their eyes by being sensitized intraperitoneally with ovalbumin (1.8 mg/kg) and aluminium hydroxide (90 mg/kg) (OVA/AH) at pH 7.4 on day 1, 8, 15, then the sensitized rabbits were challenged with 100 μl of OVA eye drops (4 mg/ml in PBS) daily for seven days, conjunctivitis was created after that rabbit divided into five groups [3, 36].

- Group I: normal group (normal group)
- Group II: Positive control (Diseased group)
- Group III: Treated by the KF suspension
- Group IV: Treated by KF- SP
- Group V: Treated by KF-SP loaded PVA/PVP MNs patch.

**Fig. 1:**
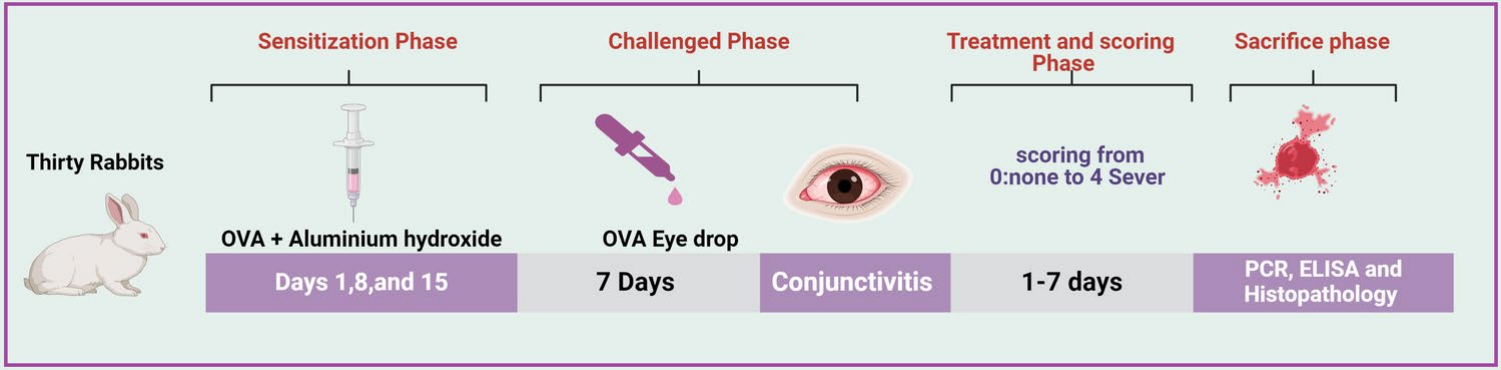
Graphical of in vivo experimental design

The treatment lasted for seven days, and finally, on day seven, AC signs and symptoms were scored based on four independent parameters, (discharge, redness, chemosis, and tearing). Each characteristic was assigned a value ranging from 0 (none) to 4 + (severe), with the total score reaching a maximum of 20 + Symptom score variations were computed and illustrated graphically as shown in Fig. 1. Animals were sacrificed after the pharmacodynamic study, conjunctiva corneal tissue was cleaned and fixed with 10% formal saline for a day after using regular saline for the histopathology study [37].

## RNA extraction and real-time PCR method

### RNA extraction and reverse transcription

The extraction of total RNA is done by the manufacturer's standard procedure by using the RNeasy Mini Kit (Catalogue no. 74104). Ethanol (96%, Applichem) was diluted to 70% with deionized water (DDW) and ß-Mercaptoethanol (Sigma Aldrich) used for extraction. Once 30 mg of tissue samples were homogenized in Buffer RLT with 10 µl/ml of ß-Mercaptoethanol using a TissueLyser (Qiagen), the samples were centrifuged, and the RNA was extracted by purification using RNeasy spin columns. Following the elution of RNA in water without RNase, any remaining DNA was eliminated by on-column DNase digestion. The reverse transcription of 1 μg of total RNA was performed in a two-step process utilizing RevertAid Reverse Transcriptase (Thermo Fisher, Cat. No. EP0441) and random hexamer primers. Additional complementary DNA (cDNA) was produced and used as a template for further quantitative real-time polymerase chain reaction (qRT-PCR) research [38].

### Gene expression assay using qRT-PCR

Gene expression levels of IGF1, Annexin A1, and Bcl2 were quantified using the Quantitect SYBR Green PCR Kit (Cat. No. 204141, Qiagen) in combination with specific primers as shown in Table 4 for sequences. Two-and-a-half microliters of 2 × QuantiTect SYBR Green PCR Master Mix, 0.5 µl of forward and reverse primers (equal to 50 picograms each), 0.25 µl of reverse transcriptase, 8.25 µl of RNase-free water, and 3 µl of complementary DNA were used for each reaction [39, 40]. The Stratagene MX3005P real-time PCR machine was used to do an IGF1, Annexin A1, and Bcl2 qRT-PCR study. In the trial for the cycling process, reverse transcription took place at 50 °C for 30 min, and then the DNA was first denatured at 94 °C for 15 min. 40 cycles of amplification were performed, each cycle consisting of denaturation at 94 °C for 15 s, annealing at 60 °C for 30 s, and extension at 72 °C for 30 s. A last extension step at 72 °C for 5 min concluded the operative process. Gene expression was standardized to Ubiquitin 5 as a reference gene by the 2 − ΔΔCt technique. A duplicate run of each sample was conducted, and the Ct values were analysed using the Strata gene MX3005P software. Comparisons were made between the amplification curves and threshold cycle values (Ct) of the experimental group and the control group to assess the relative changes in gene expression [41].

**Table 4:** List of primer sequences used for qRT-PCR

Reference	Primer sequence (5'−3')	Primer direction	Gene
**IGF1**	Forward	CCCTCTGCTTGCTCACCTT	[40]
	Reverse	TACATCTCCAGCTCCTCA	
**Annexin A1**	Forward	CCGTGCTGGAACTCGCCATAA	Designed in the current study
	Reverse	TTGGCAAAGAGACACACCATACT	
**Bcl2**	Forward	GGGGCTACGAGTGGGATGC	[39]
	Reverse	GCGGTAGCGGCGGGAGAAGT	

qRT-PCR: quantitative real-time PCR, IGF1 (Insulin-like Growth Factor 1), Annexin A1,and B-cell Lymphoma 2

### ELISA (Enzyme-linked immunosorbent assay)

Rabbits's blood was extracted by ocular extraction and placed in a 2 mL EP tube. The serum was extracted using centrifugation (2,000 × g/min for 10 min) after 8 h. Next, according to each manufacturer's recommendations, the concentrations of transforming growth factor-β (TGF-β) immunoglobulin E (IgE), interleukin-10 (IL-10), tumour necrosis factor-α (TNF-α), and interleukin-6 (IL-6) in rabbits’ serum or cell medium supernatant were measured [19].

## Histological examination of rabbits’ eyes investigation

The tissue of the cornea and conjunctiva were washed with double-distilled water and desiccated with alcohol. After soaking in molten paraffin wax, the specimens were consolidated in blocks and stored at 56 °C for 24 h. Histopathology abnormalities were investigated microscopically using a sliding microtome (Leica Microsystems SM2400, Cambridge, UK), deparaffinizing, and staining with haematoxylin and eosin.

## Statical analysis

One-way ANOVA, Tukey–Kramer multiple-comparison tests, and Student's t-test were used in the statistical analysis for the stability and comparison studies. among various groups. GraphPad Prism software version 9 was utilized to compare several groups utilizing the student's t-test at *P* < 0.05. Each result was presented as means ± SD.

## Results and discussion

### Effect of formulation variables on particle size

**Fig. 2:**
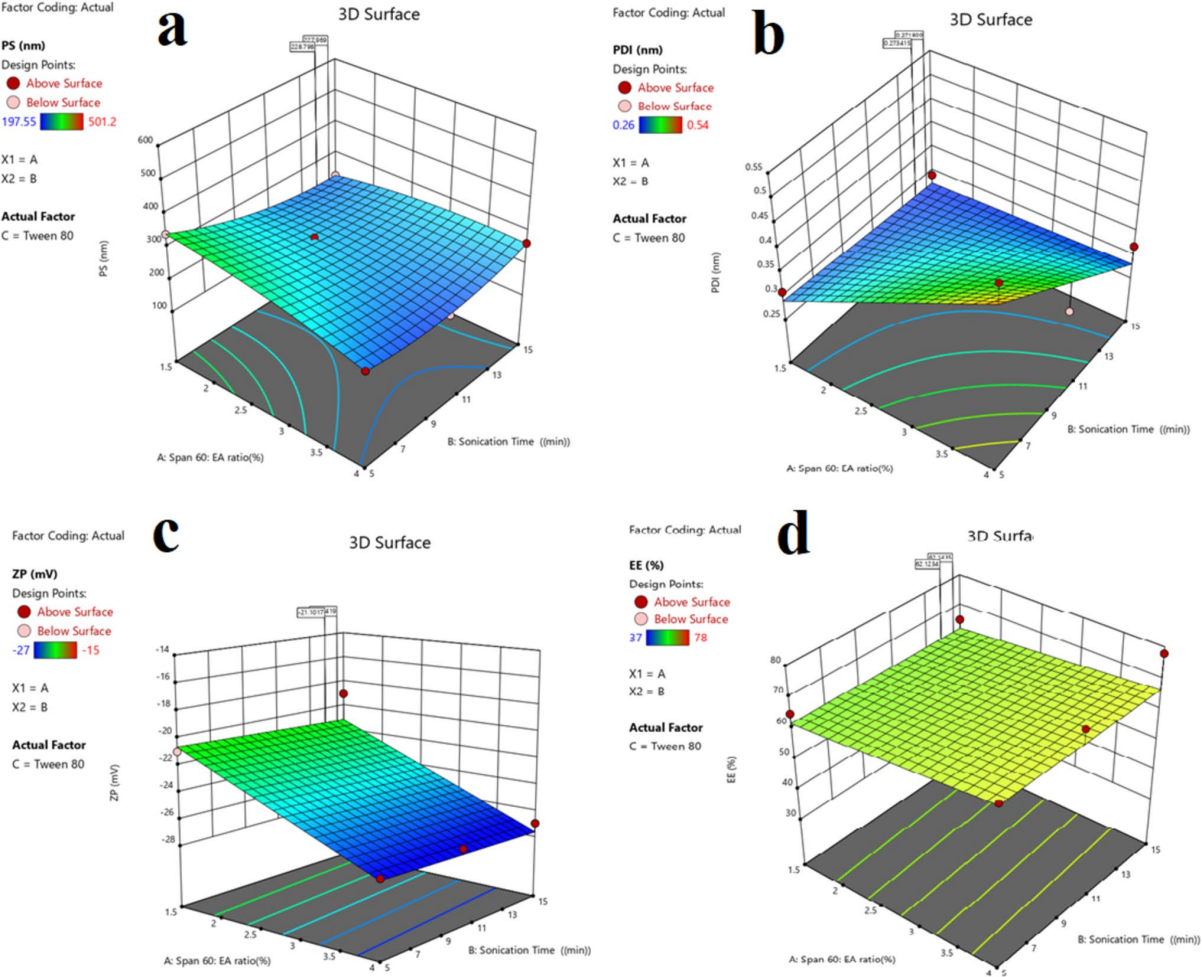
Response 3D plots for the effect of (**X1**): span to edge activator ratio, (**X2**): type of edge activator, and (**X3**): stirrer time, on (**a**) particle size, (**b**), polydispersity index (**c**) zeta potential, and (**d**) entrapment efficiency %

The stability, bioavailability, and release profile of nanosystems depend much on the particle size. Table 2 displays the PS, ZP, and PDI outcomes for the formulated Ketotifen fumarate-spanlastics. The mean particle size (PS) varied from 213 nm to 501.12 nm, indicating the average hydrodynamic diameter of the nanovesicles (Z-average). The linear polynomial equation for mean PS indicated that elevated Span 60 concentration resulted in a substantial rise in PS (*p* = 0.0046), whereas a larger concentration of the edge activator (EA), such as Tween 80, considerably decreased PS (*p* = 0.0017). The interaction between Span 60 and EA had a non-significant unfavourable effect on PS (*p* = 0.8474). Figure 2a demonstrates that a higher concentration of Span 60 led to an increased particle size (PS) due to elevated viscosity, which impeded the effective dispersion of the aqueous phase into the organic phase. Conversely, elevated concentrations of EA, especially Tween 80, decreased interfacial tension, hence promoting the generation of smaller nanovesicles. These results correspond with prior research on analogous formulations [4, 16].

## Effect of formulation variables on PDI

The polydispersity index (PDI) values for the formulations ranged from 0.26 to 0.54, indicating a rather homogenous distribution of particle sizes. The linear polynomial equation for PDI revealed that Span 60 concentration and EA had no significant effect on PDI (p-values of 0.4759 and 0.9004 respectively) as shown in Table 2. As a result, adjusting the ratio of the two components is critical for maintaining a consistent distribution of particle sizes throughout the formula. Smaller PDI values suggest more homogeneous nanovesicle sizes, which enhances physical stability and decreases aggregation as shown in Fig. 2b.

## Zeta potential

Zeta potential (ZP) values of the formulated nanovesicles ranged from −15 mV to −27 mV (Table 2). ZP determines the overall surface charge and stability of the nanovesicles. The linear polynomial equation for ZP indicated that both Span 60 (*p* = 0.0506) and EA concentration (*p* = 0.0102) significantly influenced ZP. High Span 60 concentrations imparted more negative charges, most likely due to the presence of carboxyl groups in Span 60, but EAs such as Tween 80 provided steric stability by building a mechanical barrier that inhibited aggregation [42]. Although certain formulations had lower ZP values, stability was maintained by steric hindrance, as found in prior research on similar nanovesicle systems as shown in Fig. 2c.

## Encapsulation efficiency (EE%)

The KF-SP, have entrapment efficiency (EE%) ranging from 37 to 73% (Table 2). Achieving maximum therapeutic efficacy requires a certain percentage of medication to be successfully encapsulated within the nanovesicles; this percentage is denoted as the EE%. The linear polynomial equation for EE% indicated that drug entrapment was significantly influenced by the type of edge activator (EA) and the concentration of Span 60. The increased ability of the lipid bilayer to encapsulate hydrophobic drug molecules likely accounts for the association between higher EE% and elevated Span 60 concentrations (*P* < 0.05). In the improved formulation, Tween 80 exhibited the best encapsulation efficiency at 73% among the emulsifying agents evaluated. Brij and Kolliphore RH40 secured second and third places, respectively. The improved trapping was likely facilitated by Tween 80's capacity to lower surface tension and enhance medication-vesicular membrane contact. The minimal percentage of emulsifying agent (EE%) was seen with Brij, perhaps due to its reduced solubilizing capacity [22]. Figure 2d illustrates the collaborative effect of Span 60 and EA on enhancing EE% through the 3D surface map. The most efficacious approach to drug entrapment involved a well-proportioned amalgamation of Span 60 and EA, particularly Tween 80. Consistent with earlier studies, our findings show that similar vesicular systems benefit from higher surfactant concentrations for improved drug solubilization and trapping. To achieve a high extended-release and sustained therapeutic efficacy of KF in the treatment of allergic conjunctivitis, it is essential to alter the Span 60:EA ratio.

## Investigation of factorial design

Box-Behnken statistical design was employed and statistically analyzed to assess the influence of specific variables on the attributes of the generated SP, the responses and independent variables for all SP formulations shown in Table 2. This is due to its perceived suitability as an analytical technique. The levels and factors in this publication were carefully selected after numerous trials to finalize the potential configurations of independent variables. It shows nine experimental formulations (F1-F13) that mix two components with three levels. The ratio of signal to noise is the measure of adequate precision. As indicated in Table 2, a ratio > 80:20 was obtained in each of the replies. The anticipated R^2^ also influences the quality of the model. All considered responses indicate the necessity for a satisfactory correlation (R^2^) between the adjusted and anticipated values.

The optimization process revealed that the ideal Span 60 to edge activator (Tween 80) ratio was 75:25. This ratio was determined through software analysis based on the responses of particle size (PS), polydispersity index (PDI), zeta potential (ZP), and encapsulation efficiency (EE%). The optimized formulation was further validated using dynamic light scattering (DLS) measurements, which confirmed a PS of 232.5 ± 1.9 nm, a PDI of 0.29 ± 0.01, a ZP of –28 ± 0.51 mV, and an EE% of 73 ± 0.02. These results highlight the robustness of the selected optimized formula (O.F) Span 60 to Tween 80 ratio in achieving desirable physicochemical properties essential for effective drug delivery.

## In vitro characterization of optimized formula spanlastics

### Morphological Assessment Utilizing Transmission Electron Microscopy (TEM)

**Fig. 3:**
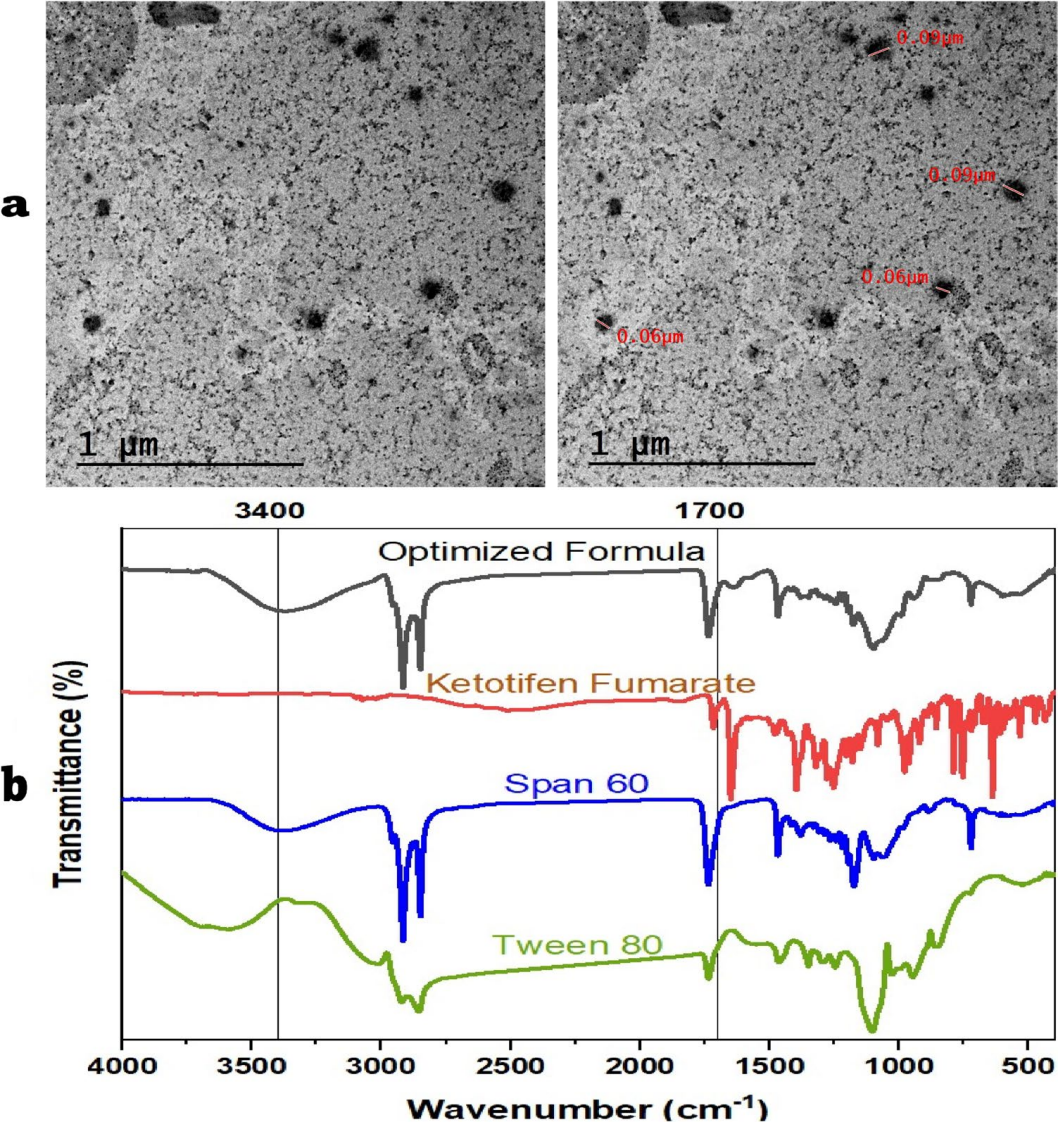
**(a) **Transmission electron micrograph and **(b)** Fourier transform-infrared spectroscopy for optimized spanlastics formula

The size, shape, and surface morphology of the nanoparticles were seen using transmission electron microscopy (TEM) (Fig. 3a). The KF-SP had consistent sizes, morphology revealed a small, round, nanometer-sized particle with no signs of aggregation.

### Fourier transform-infrared spectroscopy (FT-IR) analysis

Figure 3b shown FTIR spectra of KF, excipients (Span 60, Tween 80), and the optimized SP to see chemical stability and interaction. The FTIR spectrum of pure KF has peaks around 3400 cm^⁻1^, since these peaks show the O–H stretching wave, the chemical has a hydroxyl group. The main peak at 1700 cm⁻^1^ is caused by the C = O stretching in the fumarate part. Both Span 60 and Tween 80 have their specific peaks that are caused by the chemicals that make them up. The peaks in Span 60 are around 2915 cm⁻^1^ and 2849 cm⁻^1^, which show that C-H is stretching. By contrast, C-H bending waves produce the bands at 1465 cm-^1^. Tween 80 has aliphatic chains shown by comparable peaks [43, 44]. The FTIR analysis reveals that KF was effectively added to the combination since the special peaks of the medication were kept. However, small changes in the peaks suggest that the drug may have physical interactions with Span 60 and Tween 80. The fact that there are no new peaks shows that the excipients are chemically compatible and that the drug's functional groups are still intact. This means that the formulating process has not changed the structure of the drug. This showed that KF did not react chemically with the excipients because there were no clear functional group spots.

### Differential scanning calorimetry (DSC) Analysis

**Fig. 4:**
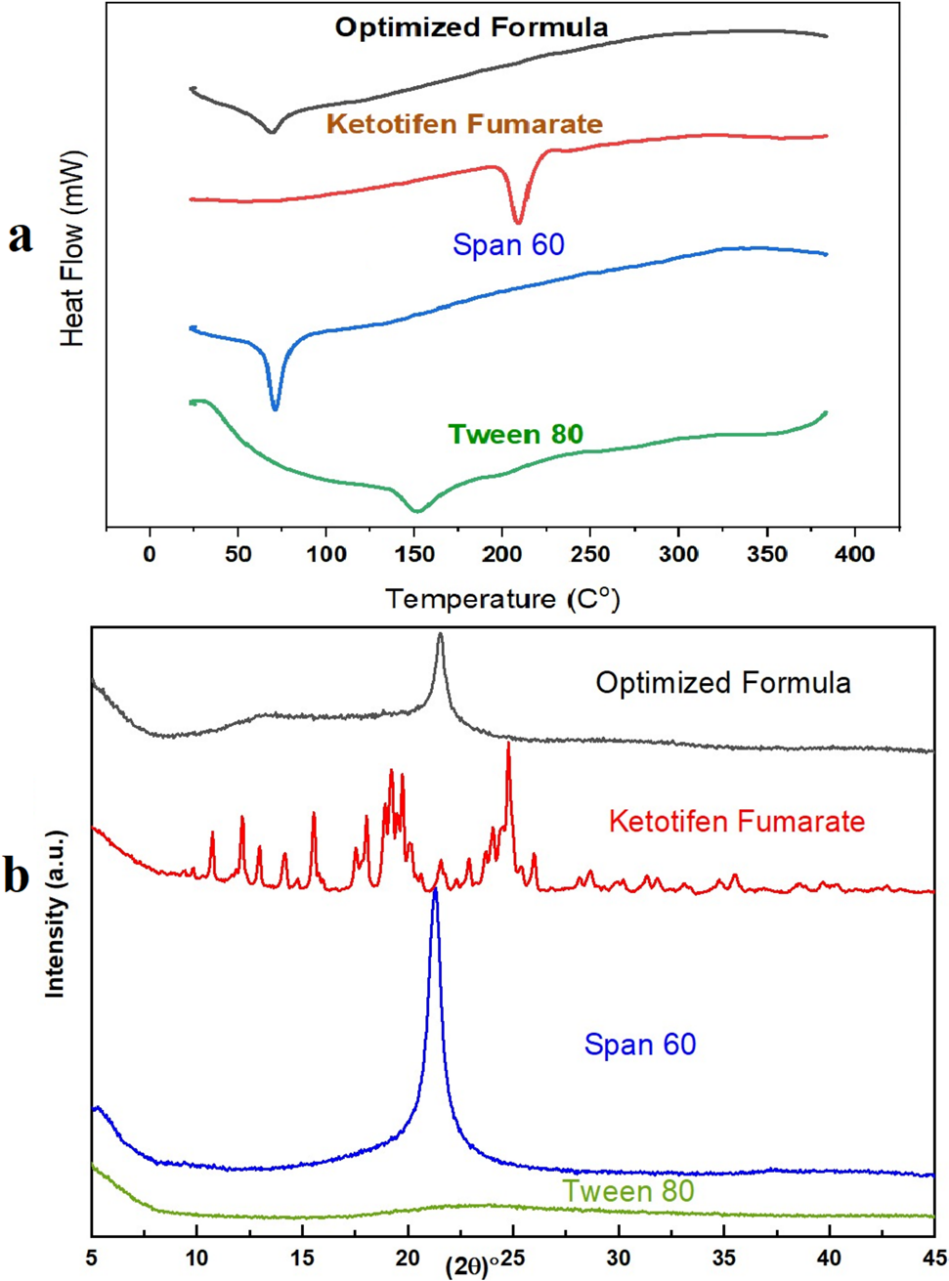
**(a)** Differential scanning calorimetry (DSC) and **(b)** X-ray diffraction (XRD) for optimized formula, Ketotifen fumarate, span 60 and tween 80

The DSC thermogram profiles of pure KF, excipients (Span 60, Tween 80), and the ideal SP formulation are shown in Fig. 4a. We investigate with these profiles possible interactions and thermal behaviours [21]. The molecule melts at 142 °C and has a solid structure based on the endothermic peak of Pure KF's thermogram at this temperature. Span 60's melting transition is shown on its thermogram with a clear endothermic peak at 55 °C. The melting transition of Span 60 is shown by a prominent endothermic peak at 55 °C in its thermogram. The thermal transition is semi-crystalline, and the peak is distinctly delineated, signifying stability under experimental conditions [17]. Tween 80 has an extensive endothermic transition at 195 °C attributable to its amorphous configuration and melting components. Amorphous surfactants exhibit wider peaks. The thermogram of the optimized SP markedly contrasts with the components. The prominent endothermic melting peak of KF at 142 °C is no longer observable, indicating that the substance has transitioned from a crystalline to an amorphous or molecularly dispersed state inside the SP matrix [16]. Span 60 and Tween 80's thermal characteristics are substantially unchanged, indicating that the excipients' structural integrity was preserved throughout the preparation procedure. The DSC analysis, which shows that pure KF has a distinct melting point of 142 °C, confirms that the material is crystalline. The absence of the peak in the improved SP formulation suggests that the medication is either amorphous or molecularly dispersed within the matrix, as it is no longer crystallized. The thermal stability of Span 60 and Tween 80 is maintained by maintaining their transitions [20, 45].

### X-ray diffraction (XRD) analysis

Figure 4b shows the XRD of KF crystallizes, along with the added substances (Span 60 and Tween 80) and the best mixture. XRD patterns of pure KF show clear, noticeable peaks, which means that it has crystallized. Pure medicines have ordered crystal structures that make unique designs. There are a lot fewer crystalline peaks and broad designs in Span 60 and Tween 80. Span 60 has a single peak, which means it is semi-crystalline, while Tween 80 is mostly amorphous. As surfactants, these ingredients keep the end mixture stable and might change the crystal structure of the drug. The XRD pattern of the improved formula shows a clear decline in the drug's characteristic peaks, pointing to a partially crystalline or amorphous structure [46]. This signifies effective drug encapsulation or dispersion within the excipient matrix, perhaps enhancing solubility and bioavailability by restricting crystallization.

XRD analysis indicates that the amended formula reduced the crystallinity of KF, hence enhancing its solubility and dissolving rate. The absence or reduction of pronounced peaks in the adjusted formula indicates improved drug distribution within the excipient matrix. Given that crystalline drugs dissolve at a slower rate than their amorphous counterparts, this finding is advantageous for formulations aimed at enhancing solubility and bioavailability. Span 60 and Tween 80 alter the crystalline structure, with Tween 80 significantly enhancing the amorphous state, hence improving medicine solubility [16].

### Short stability study of ketotifen fumarate spanlastics

Stable Spanlastics are essential for their potential as an alternative to established delivery techniques. The short-term stability of the Optimized SP formula with high drug entrapment efficiencies was examined over three months. Table 5 displays variations in PS, PDI, ZP, and %EE of SP at 4 °C over three months. To be stable, an SP formulation must retain consistent PS, PDI, ZP, and %EE, and avoid phase separation or colour changes throughout storage. The short-term stability results of the optimized formula at 4 °C and 25 °C for 3 months [9]. PS, PDI, ZP and %EE were studied as stability parameters, results are represented by mean ± SD (n = 3).

**Table 5:** The short-term stability results of optimized spanlastics at 4 °C and 25 °C for 3 months. mean ± SD (n = 3)

Parameters	KF-SP Freshly Prepared	KF-SP After Three Months of Storage at 4 °C	KF-SP After Three Months of Storage at 25 °C
PS (nm)	232.5 ± 1.9	229.23 ± 0.52	230 ± 0.21
PDI	0.29 ± 0.01	0.30 ± 0.32	0.29 ± 0.21
ZP (mV)	−28.3 ± 0.51	−28.41 ± 0.01	−28.24 ± 0.01
EE (%)	74 ± 0.43	71 ± 0.31	72 ± 0.62

KF-SP: Ketotifen fumarate loaded spanlastics

### Physical characteristics of the Microneedles

Demonstrates that optimal ocular penetration and reduced discomfort require microneedle length (M1: 243 ± 0.15 µm, M2: 250 ± 0.10 µm, and M3: 261 ± 0.05 µm). M3 is the longest, than M2 and M3. This indicates that drug loading weakens microneedles by lengthening them. The tip diameters are: M1 (13. ± 0.04 µm), M2 (17. ± 0.21 µm), and M3 (15. ± 0.01 µm). M1, M2, and M3 have the smallest tip diameters, which influences penetration and insertion. This suggests that M1 may be less visible than M2 and M3, which can transport medication. The base diameters are: M1 (104 ± 0.56 µm), M2 (108 ± 0.12 µm), and M3 (104 ± 0.32 µm) as shown in Table 6 [12]. The diameter of the microneedle's base indicates its mechanical stability; larger bases indicate better structure. M2 has the largest base diameter, followed by M1 and M3, which suggests a PVA-based relationship. Microneedles must be strong enough to pass through the ocular layer without breaking. Mechanical resistance is indicated by a height drop under forces of 250 g, 500 g, and 1000 g. M1: All forces cause moderate height loss, indicating increased mechanical strength due to higher PVA content.M2 has comparable mechanical performance to M1, but with less height loss when forced.M3, with the greatest height loss, may have lower mechanical strength due to the low PVA content. M1, M2, and M3 each contain 53%, 76%, and 61% KF microneedles. KF's higher drug content improves therapeutic dosage. The minority of medication is in M3, but mechanical strength and dissolution may suffer. The mechanical performance of microneedles is measured by the height decrease (µm) under applied forces (250, 500, 1000 g). M1's mechanical reaction and height reduction remain consistent across stresses. M2 falls less than M1, indicating high mechanical integrity, whereas M3 falls the most, indicating low mechanical strength. Formulation M2 is the most balanced among M1, M2, and M3 for a variety of reasons. The 10% KF drug content in M2 strikes a balance between therapeutic efficacy and mechanical integrity. The mechanical strength of M2 supports drug delivery and eye penetration. The M1 microneedle has the smallest tip diameter, but the M2 has a suitable length, tip diameter, and base diameter. M2 (10% KF, 10% PVA, 25% PVP) is the best formulation for developing KF-loaded MNs for ocular administration because of its balanced pharmaceutical concentration, mechanical strength, and microneedle sizes, which may improve conjunctivitis treatment [33].

**Table 6:** Physical characteristics of the Microneedles

MNs	Length (µm)	Tip Diameter (µm)	Base Diameter (µm)	Drug content (%)	Length after Forces Applied per Array (µm)		
					250 gm	500 gm	1000 gm
M1	243 ±f0.15	13 ± 0.04	104 ± 0.56	53%	237 ± 0.41	210 ± 0.15	200 ± 0.13
M2	250 ± 0.10	17 ± 0.21	108 ± 0.12	76	247 ± 0.34	245 ± 0.45	243 ± 0.53
M3	261 ± 0.05	15 ± 0.01	104 ± 0.32	61	255± 0.23	247 ± 0.12	230 ± 0.15

MNs: Microneedle; M1:microneedle containing 5% ketotifen fumarate,15% Polyvinyl Alcohol, and 20% Polyvinylpyrrolidone, M2: microneedle containing 10% ketotifen fumarate,10% Polyvinyl Alcohol, and 25% Polyvinylpyrrolidone and; M3: microneedle containing 15% ketotifen fumarate,5% Polyvinyl Alcohol, and 30% Polyvinylpyrrolidone

## Characterization of optimized Microneedle

### Scanning electron microscopy (SEM)

**Fig. 5:**
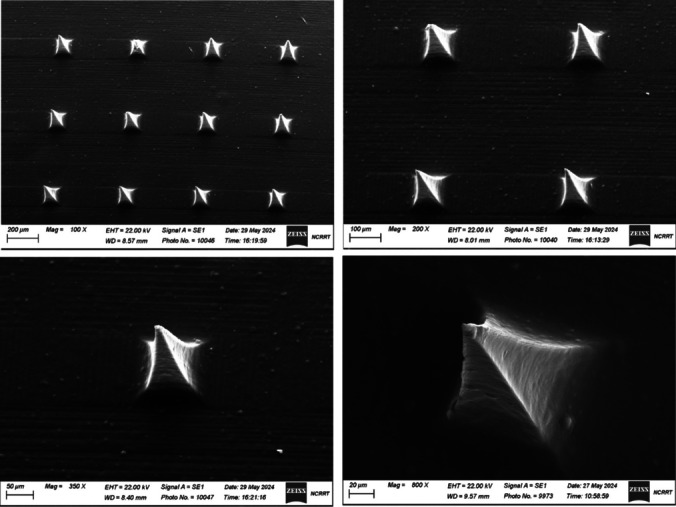
Scanning electron microscopy of optimized PVA/PVP-microneedle loading freeze-dried Ketotifen fumarate-loaded spanlastics (M2)

Figure 5 shows SEM images of KF-SP-loaded PVA/PVP microneedles for ocular conjunctivitis. Microneedles are popular in ocular medicine because they are less invasive. SEM images show the revolutionary ocular system's construction and efficacy. 100X and 200X magnification reveal well-organized microneedles with consistent spacing and size distribution. Continuous patch drug administration and proper eye contact necessitate homogeneity. As shown in the 350X and 800X images, ocular microneedles must be sharp enough to pierce the conjunctival membrane without causing injury. The smooth surface and absence of microneedle cracks or deformation suggest structural integrity in the PVA/PVP mix with KF-SP. This blend is both biocompatible and mechanically stable for transdermal and ocular medication delivery [43, 47]. Microneedles' sharp points allow them to transport drugs precisely to the conjunctiva without causing patient pain, as evidenced by higher magnification photos. The nanoscale SP system in microneedles may maintain KF release, which improves AC treatment. SEM analysis confirms that KF-SP-loaded PVA/PVP microneedles can deliver ocular medications. Microneedles can gently penetrate the eye thanks to their sharp, well-defined tips and consistent morphology. Their smooth, polished surfaces and lack of flaws suggest high-quality materials that stabilize and regulate drug release. Microneedle technology may help conjunctivitis patients adhere to their treatment plans and administer KF more precisely [48].

### Fourier transform infrared (FTIR) Analysis

**Fig. 6:**
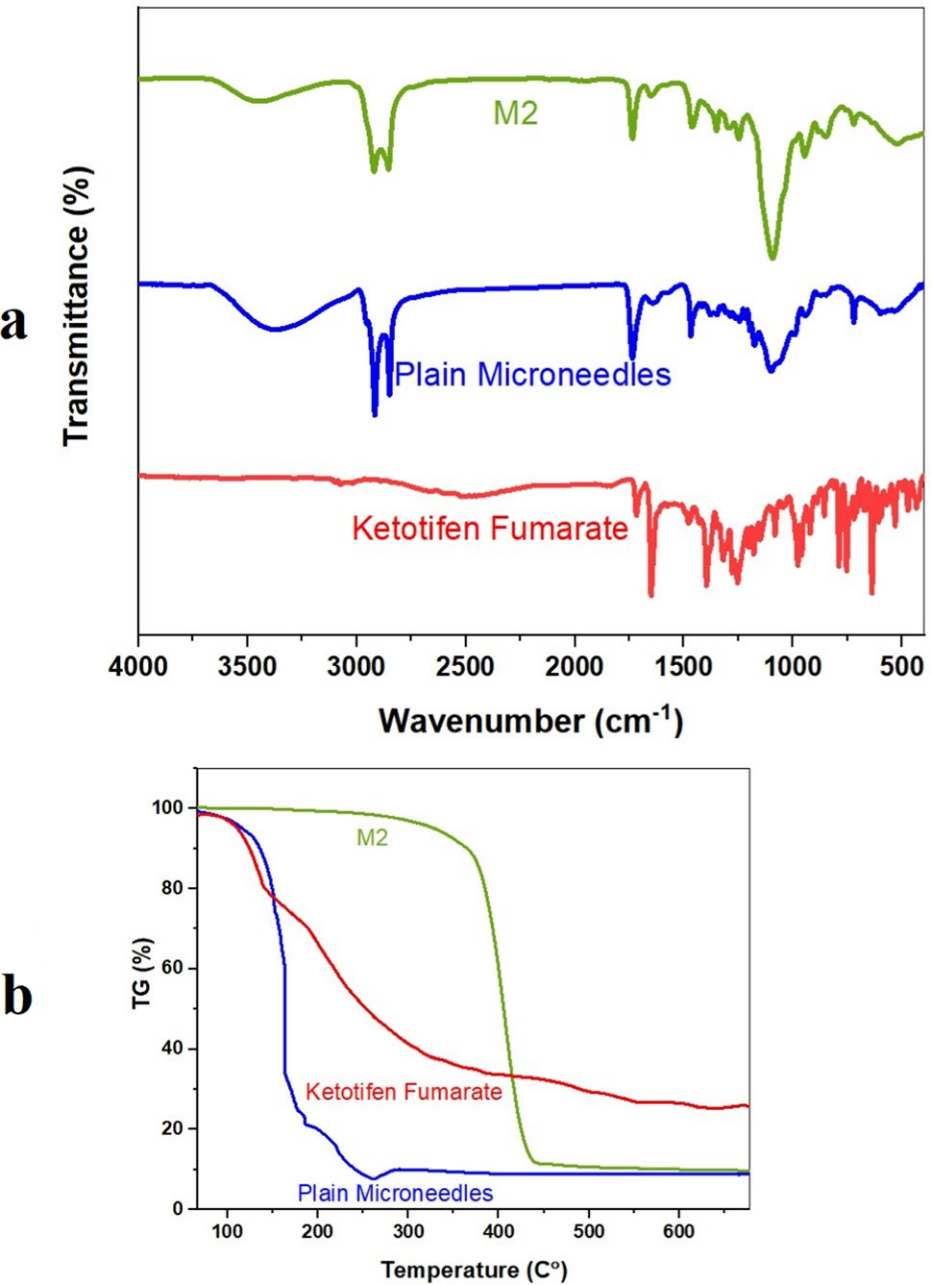
In vitro*,* release from various developed Ketotifen fumarate (KF) suspension, optimized formula of ketotifen spanlastic (KF-SPs), and optimized PVA/PVP-Microneedle loading freeze-dried Ketotifen fumarate-loaded Spanlastics (M2). Note: **** indicates significance between data with p value < 0.0001

Figure 6a shown the FTIR spectra of KF functional groups, plain microneedles, and optimized formula M2. Chemical interactions and formulation-induced molecular structural changes are detected using FTIR. The FTIR spectrum of pure KF contains several functional group peaks. A peak at 3430 cm⁻^1^ indicates the N–H stretching of secondary amines. The peak at 1650 cm⁻^1^ indicates amide group C = O stretching. Peaks at 1400–1600 cm⁻^1^ indicate aromatic C = C stretching, while those below 1000 cm⁻^1^ indicate C-H bending and other interactions [20, 49]. The absence of KF FTIR peaks in plain microneedles indicates that the matrix is medication-free. O–H stretching at 3200–3600 cm⁻^1^ indicates polymers like PVA or PVP in microneedles, while weaker peaks near 1700 cm⁻^1^ reveal ester or carbonyl groups in the matrix. A decrease or shift in KF's characteristic peaks in the M2 spectrum suggests encapsulation or interaction with the microneedle matrix. KF's strong O–H stretching at 3400 cm⁻^1^ may overshadow the N–H stretching. A small carbonyl peak at 1650 cm⁻^1^ indicates the presence of the drug in the formulation, though modified. According to FTIR, KF was successfully incorporated into the M2 microneedle formulation. Drug peaks decreased or disappeared, indicating molecular interaction. The drug's characteristic peaks are missing or shifted in microneedle spectra, indicating hydrogen bonding with the polymer matrix. KF may be encapsulated in microneedles. Amorphization may improve drug solubility and bioavailability in drug delivery systems because the M2 spectrum lacks KF peaks. Microneedles or polymer matrices have been shown in studies to improve the solubility and absorption of amorphous drugs [23, 24].

### Thermogravimetric Analysis (TGA)

Figure 6b shown the thermogravimetric analysis (TGA) of KF, the heat stability and decomposition curves for KF, Plain Microneedle, and M2 Microneedle TGA. KF loses weight after 100 °C water loss. Drugs degrade at temperatures ranging from 300 to 500 degrees Celsius. Heat causes organic molecules to break down. Simple microneedles enable two-phase weight loss. At 100–150 °C, moisture or solvent evaporation causes the weight of the microneedle matrix to decrease. Microneedles made of PVA and polyvinylpyrrolidone decompose at temperatures ranging from 200 to 400 °C [34].

The polymer matrix of uncoated microneedles completely liquefies at 450 °C, rendering them less thermally stable than the drug. M2 microneedles have the same thermal profile as basic models, but with a slight deterioration shift. At 100 °C, microneedles cause moisture and weight loss. M2 degrades at temperatures higher than regular microneedles, between 250 °C and 400 °C. High temperatures are more stable with KF, which slows decline. At 450 °C, M2 and microneedle materials degrade completely [45]. TGA results show that M2 microneedles are more stable at high temperatures than standard ones. KF in the polymer matrix inhibits degradation, demonstrating its stability. Because of its improved thermal stability, the drug-polymer system withstands high temperatures. This makes it easier to prepare and store the microneedle mixture. TGA demonstrates that M2 microneedles hold medicine while retaining polymer structure. Regular microneedles lose weight more gradually than M2. As a result, the medicine has been encapsulated and stabilized [50].

## In vitro drug release and pharmacokinetics study

**Fig. 7:**
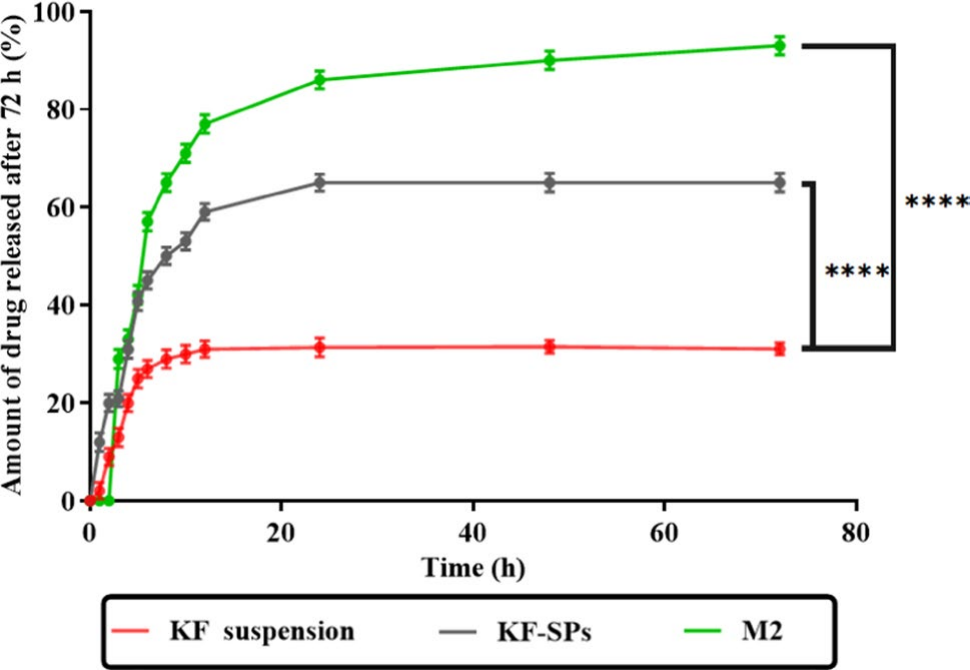
Fourier transform infrared (FTIR) analysis and thermogravimetric Analysis (TGA) for Ketotifen fumarate suspension, plain microneedle (without drug), and optimized microneedle formula (M2)

Figure 7 shows the cumulative percent release of KF from KF Suspension during an in vitro drug release and pharmacokinetic study. The first few hours see burst release, which increases to 20% after four hours. After the initial burst, there is no further release for 24 h, with a plateau at 31%, providing a quick start to action. It must be taken regularly to keep therapeutic levels stable [16, 51]. Compared to suspension, KF-SPs release KF more slowly. A steady increase in medication release over 72 h, peaking at 65% after 24 h. prolongs the action, reduces dosage frequency, and boosts patient compliance. Similar to KF Suspension, release increases and lasts longer, with significant variation after the initial burst phase. KF release from KF-SP-loaded PVA/PVP MNs was delayed for the first two hours, most likely due to microneedle disintegration and microchannel formation. Release increased quickly, reaching 29% after three hours and 93% after 72 h. The combination of continuous release and localized delivery resulted in the highest percentage of total release among the three formulations, indicating effective medication administration. KF-SPs and KF Suspension have significantly higher and longer releases than the suspension. The improved microneedle delivery system enables KF suspension to release faster and plateau earlier than KF-SPs, necessitating frequent use even for short-term relief. KF-SP-loaded PVA/PVP MNs have the best release profile an initial burst followed by steady release resulting in maximum bioavailability and a longer therapeutic effect. KF-SPs outperform KF suspension in terms of drug release and M2 by 1.6 and 2.7 times, respectively. This formulation is likely the most effective at maintaining medication levels, reducing administration frequency, and increasing patient compliance [33, 52].

Drug release from KF-SP-loaded PVA/PVP microneedles (M2), KF-SP, and KF suspension was modelled mathematically, Zero-order, First-order, Higuchi, Hixson-Crowell, Korsmeyer–Peppas models are featured. Table 7 shows the R^2^ value for each model, indicating its fit with experimental data. Zero-order kinetics shows that KF-SP-loaded PVA/PVP MNs have the highest R^2^ value (0.991), indicating a superb model fit. A steady drug release rate maintains drug levels. KF-SP shows a good match (R^2^ = 0.943), indicating a stable release with little oscillations. KF Suspension has a Moderate fit (R^2^ = 0.864) suggesting a decreasing release rate over time. KF-SP-loaded PVA/PVP MN have First-order kinetics with R^2^ = 0.912, indicating drug concentration impacts release rate. KF-SP have a lower fit (R^2^ = 0.876) suggesting limited model applicability. In KF Suspension, an excellent match (R^2^ = 0.903) suggests concentration-dependent release. Strong fit (R^2^ = 0.978) in the Higuchi Model for KF-SP-loaded PVA/PVP MNs suggests diffusion-controlled drug release. The excellent fit of KF-SP (R^2^ = 0.964) indicates diffusion control. A good match (R^2^ = 0.946) in KF suspension suggests a diffusion-based release mechanism. Hixson-Crowell Model: KF-SP-loaded PVA/PVP MNs: R^2^ = 0.952, indicating drug particle surface area and diameter changes drive release [34].

**Table 7:** Mathematical models and their correlation coefficients

Model	R^2^	KF-SP loaded PVA/PVP MNs	KF-SP	KF Suspension
Zero Order		0.991	0.943	0.864
First Order		0.912	0.876	0.903
Higuchi		0.978	0.964	0.946
Hixson–Crowell		0.952	0.929	0.911
Korsmeyer–Peppas	N	0.985 (n = 0.45)	0.972 (n = 0.55)	0.932 (n = 0.68)

KF-SP loaded PVA/PVP MNs: Ketotifen Fumarate-loaded Spanlastics incorporated into Polyvinyl Alcohol (PVA) and Polyvinylpyrrolidone (PVP) Microneedles (MNs), KF-SP: Ketotifen Fumarate, and KF Suspension: suspension of Ketotifen Fumarate

A good fit (R^2^ = 0.929) in the KF-SP model suggests particle size dependence and a moderate fit (R^2^ = 0.911) indicates model relevance for KF Suspension, but less than MNs. A Korsmeyer-Peppas Model fits well (R^2^ = 0.985) with an exponent value (n = 0.45), indicating a Fickian diffusion process for KF-SP-loaded PVA/PVP. The strong fit (R^2^ = 0.972) and exponent value (n = 0.55) suggest a non-Fickian transport mechanism in KF-SP. KF Suspension exhibits a strong fit (R^2^ = 0.932) and exponent value (n = 0.68) indicating a mix of diffusion and erosion mechanisms. The mathematical models demonstrate that KF-SP-loaded PVA/PVP microneedles (MNs) release at a controlled rate with a mostly zero-order profile. Maintains therapeutic medication levels. The Higuchi and Korsmeyer–Peppas models fit well, proving diffusion is the main MN drug release mechanism. KF-SP and KF suspension release mechanisms are more sophisticated and involve diffusion and erosion when fitted to Higuchi and Korsmeyer–Peppas models. The Zero-order model doesn't fit these formulations, implying time-dependent release rates. The regulated release profile of the KF-SP-loaded PVA/PVP MN formulation makes KF dosing in conjunctivitis effective and sustained [48]. In conclusion, the M2 formulation of KF-loaded microneedles is the best for delivering drugs to the eye because it has the right amount of drug loading, mechanical integrity, and controlled release. This formulation may make it much easier to treat eye problems like conjunctivitis by getting patients to take their medicine as prescribed and reducing the number of times they need to do it.

## In vivo study

### Evaluation of ocular signs for rabbits

**Fig. 8:**
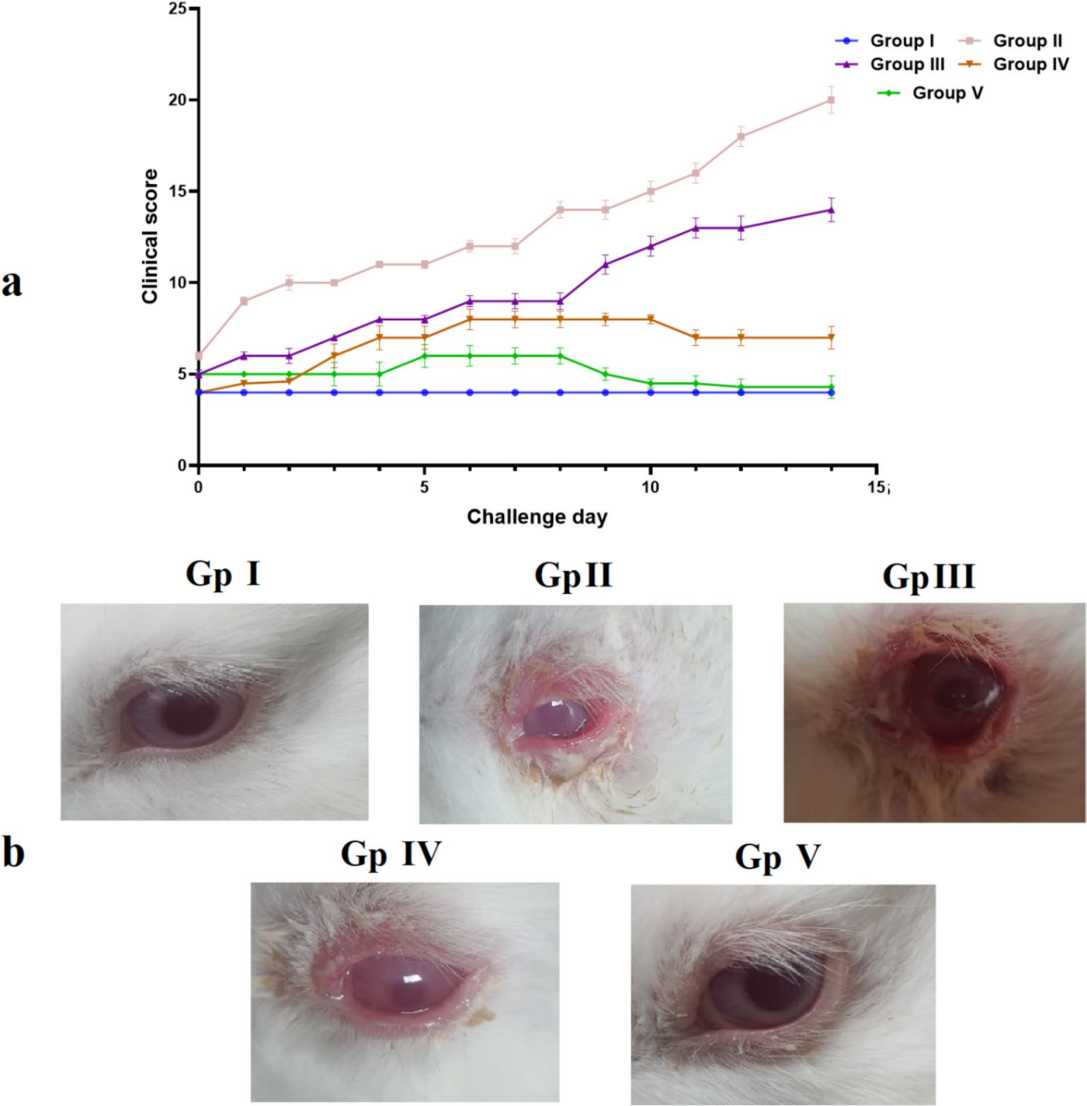
(**a**) Eye feature scores of rabbits in each group, and (**b**) ocular signs of allergic conjunctivitis in rabbits in each group. Abbreviations: Group I: normal group (normal group); Group II: Positive control (Diseased group); Group III: Treated by the KF suspension; Group IV: Treated by KF- SP; and Group V: Treated by KF-SP loaded PVA/PVP MNs patch

Figure 8 shows (a) eye feature scores of rabbits in each group, and (b) Ocular signs of allergic conjunctivitis in rabbits in each group. The results showed the eye symptoms of rabbits in Group II were Positive control (Diseased group), and Group III was treated by the KF suspension were more serious, and the score was also higher than that in Group I (normal group). Group IV was treated by KF-SP, and Group V was treated by KF-SP loaded PVA/PVP MNs patch and observed no significant change compared to Group I. The above results indicate that Group V was treated by KF-SP-loaded PVA/PVP can effectively relieve the symptoms of AC [53].

### Effect of the RNA extraction and Real-Time PCR on transcription levels

To measure the amounts of IGF1, annexin A1, and Bcl2 genes in the tissues, used qRT-PCR, which stands for quantitative real-time polymerase chain reaction. After the RNA was extracted and reverse transcription was finished, the Quantitect SYBR Green PCR Kit was used to perform quantitative real-time PCR. The housekeeping gene Ubiquitin 5 was used to normalise gene expression data. Because of this, we were able to compare the two groups, experimental and control, with precision. The findings revealed a drastic difference in the levels of target gene transcription.

**Fig. 9:**
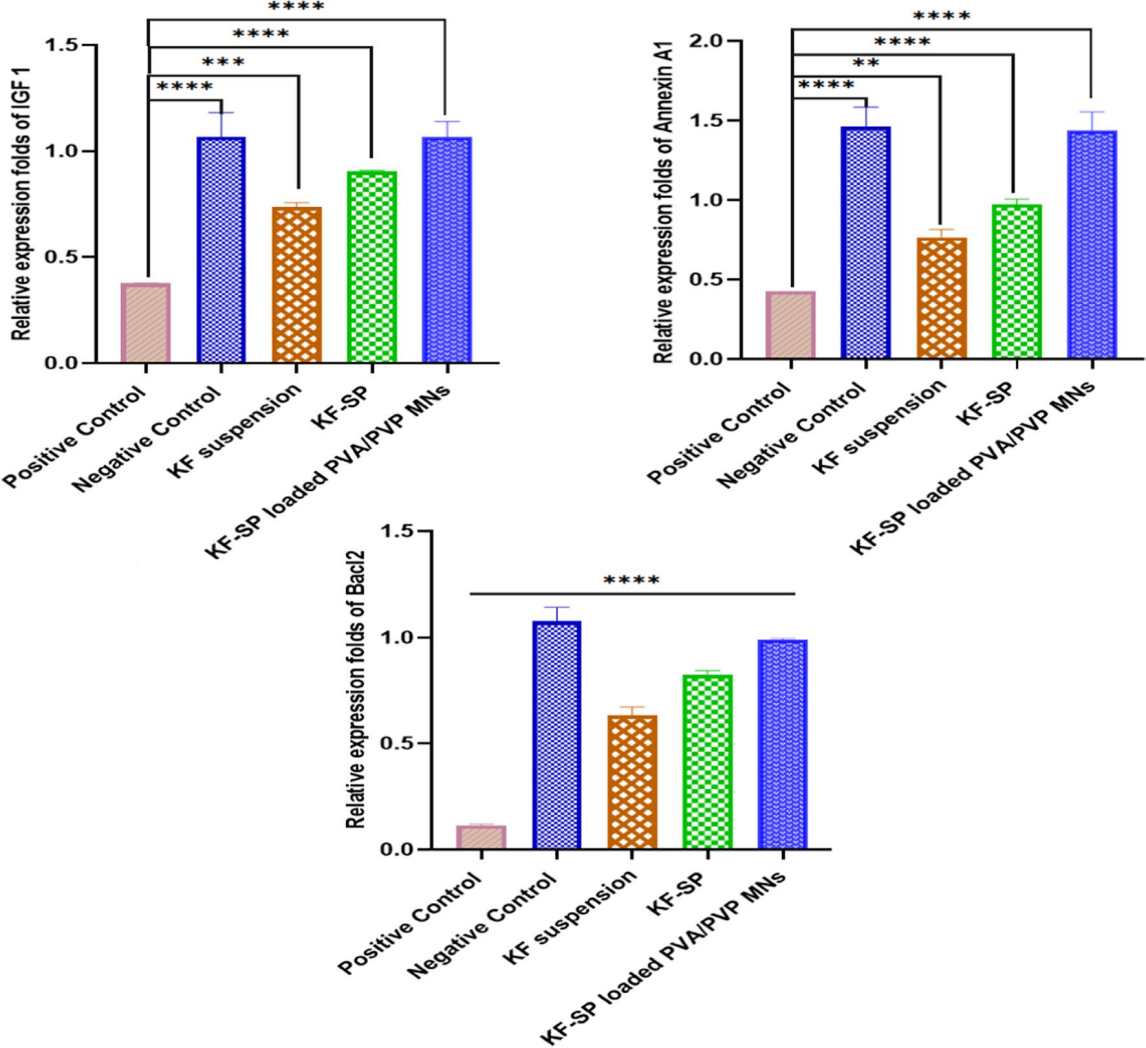
Effect of different formulations of Ketotifen fumarate on serum inflammatory cytokines protein profile; transforming growth factor-β (TGF-β) immunoglobulin E (IgE), interleukin-10 (IL-10), tumour necrosis factor-α (TNF-α), and interleukin-6 (IL-6). Note: The data are presented as means ± SE (n = 3), * = 0.0151, ** = 0.0010, **** < 0.0001, KF: Ketotifen fumarate, SP: Spanlastics, PVA/PVP MNs: Polyvinyl pyrolidine/Polyvinyl alcohol Microneedles

Significant inhibition of transcriptional activity was indicated by a marked decrease in IGF1 expression in the treated group compared to the control [54]. The experimentally measured reduction in annexin A1 expression following treatment was comparable to this, with inhibition rates of approximately X%. Bcl2, an important regulator of apoptosis, may be involved in cell survival pathways since the treatment circumstances greatly elevated its downregulation. The effectiveness of the treatment method in changing gene expression was demonstrated by the statistically significant decrease in IGF1, annexin A1, and Bcl2 in the experimental group as compared to the untreated control group (*P* < 0.05). The differential 2 − ΔΔCt analysis led to the discovery of transcription level variations, and the amplification curves that followed clearly distinguished between treated and untreated samples. Study the affected transcriptional levels of genes implicated in signal transduction pathways, cell proliferation, and apoptosis, and our results show that RNA extraction and Real-Time PCR are viable tools for assessing changes in gene expression. According to the PCR results, KF-SP-loaded PVA/PVP microneedles are superior to KF solution or KF-SP in treating inflammatory and allergic ocular conditions [55]. The improved effectiveness of the microneedle system in treating allergies and generalised ocular inflammation makes it a viable technique for the continuous, focused delivery of drugs in ophthalmology as shown in Fig. 9.

IGF-1, Annexin A1, and Bcl2 were chosen as biomarkers in this study because of their important roles in inflammatory and apoptotic pathways associated with allergic conjunctivitis. IGF-1 is a growth factor that plays an important role in cell survival and tissue repair, as well as anti-inflammatory properties that are essential for restoring ocular health. Lower IGF-1 levels are often linked to damaged cells and more inflammation, which makes them an important sign of how well a treatment is working. Annexin A1, an anti-inflammatory mediator, controls the movement of white blood cells and helps inflammation go away, directly addressing the inflammatory processes that lead to allergic conjunctivitis. Bcl2, an apoptosis regulator, protects cells by inhibiting apoptosis caused by inflammation-induced stress. The modulation of these biomarkers reveals mechanistic information about the therapeutic potential of the KF-SP-loaded microneedle system. These biomarkers effectively demonstrate how the KF-SP system's outcomes align with its intended therapeutic goals by targeting inflammation, preventing apoptosis, and improving tissue repair. Their inclusion in the analysis provides a thorough understanding of the system's impact on the molecular level.

### ELISA (Enzyme-linked immunosorbent assay)

**Fig. 10:**
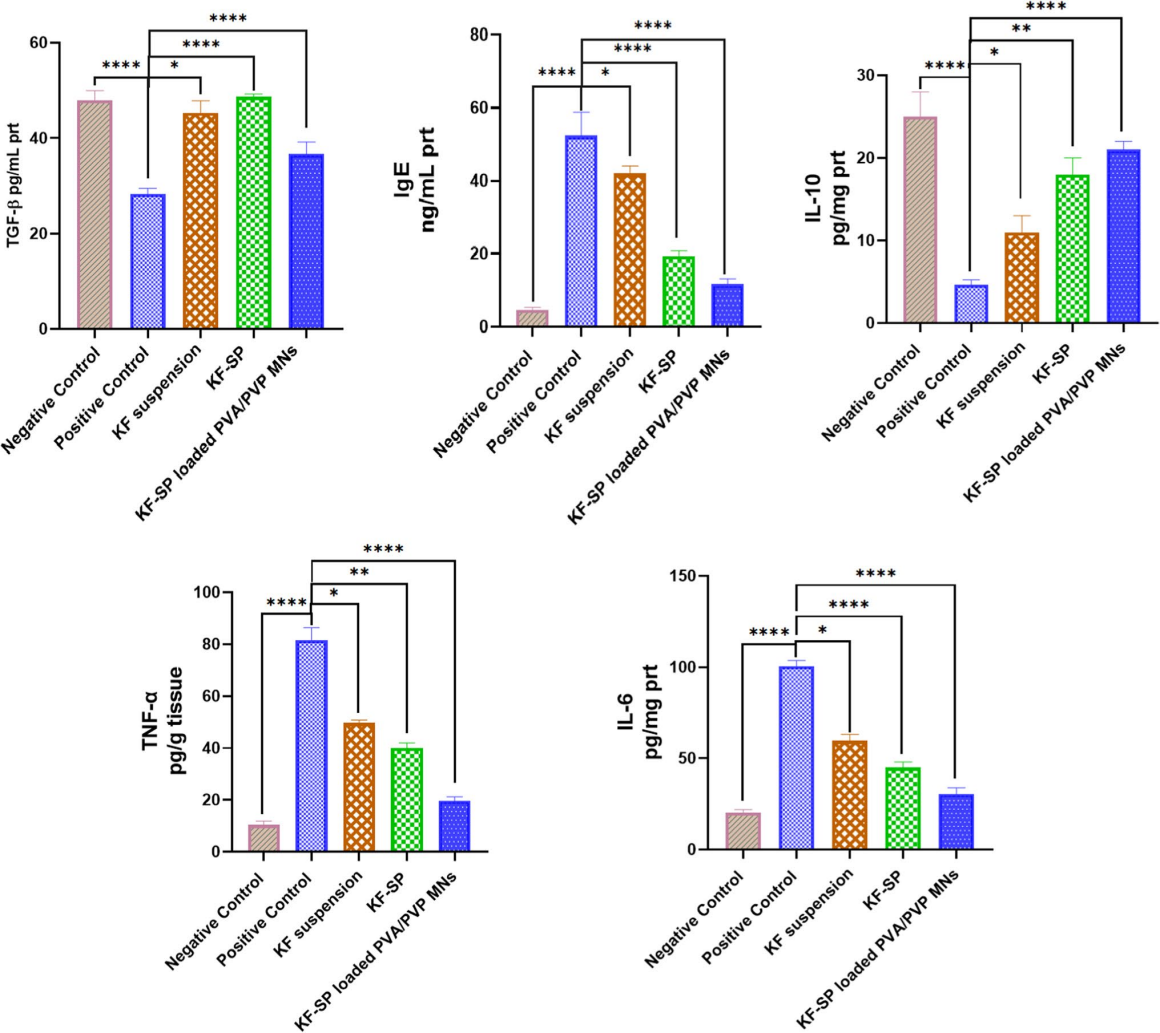
Bar chart of qRT-PCR analysis demonstrating the relative transcription levels of (**a**) IGF1, (**b**) Annexin A, and (**c**) Bacl2 genes treated with KF suspension, KF-SP, and KF-SP loaded PVA/PVP MNs patch, and compared to the positive and negative control. The error bars represent the SD of three replicates. Note: KF: Ketotifen fumarate, SP: Spanlastics, PVA/PVP MNs: Polyvinyl pyrolidine/polyvinyl alcohol microneedles. The statistical analysis was executed using one-way ANOVA followed by Tukey’s Multiple Comparisons test, (**) = 0.0036, (***) = 0.0002, and (****) < 0.0001: significance was compared to the positive control; qRT-PCR: quantitative real-time PCR.

Figure 10 shows how different KF formulations affect key inflammatory and immunological markers, particularly in rabbits exposed to allergy and inflammatory stimuli. Research indicates that standard KF suspension can regulate immunological responses, as seen by a decrease in pro-inflammatory markers (TNF-α, IL-6) and a partial increase in anti-inflammatory factors (IL-10). The small drop in IgE blood levels showed that this formulation could not minimize the allergic reaction, a significant improvement was shown with KF-SP. SP, designed for drug delivery, reduced pro-inflammatory cytokines and increased anti-inflammatory activity, improving therapeutic effects. Enhanced IL-10 levels indicated a more effective reduction of inflammation, however, reduced TNF-α and IL-6 levels were detected compared to the KF suspension group [35]. Moreover, restoring TGF-β levels to their original levels in this group indicates enhanced tissue repair and regeneration. Although KF-SP may require further improvement to achieve optimal treatment outcomes, it demonstrated greater efficacy compared to the suspension. While KF-SP may need to be enhanced to provide the best therapeutic effects, it proved to be more successful than the suspension. KF-SP-loaded PVA/PVP microneedles (M2) had the greatest anti-inflammatory and anti-allergic potency across all criteria. This new formulation reduced IgE levels close to normal, demonstrating its outstanding ability to modulate allergic responses. Loaded MNs with KF-SP showed significant reductions in TNF-α and IL-6, controlling the inflammatory process. This formulation also produced the most IL-10, boosting its anti-inflammatory properties. TGF-β restoration confirms KF-SP-loaded MNs' effectiveness in tissue regeneration and restoring normal physiological conditions.The KF-SP-loaded PVA/PVP microneedles' steady and accurate drug release ensures a long therapeutic effect. The microneedles increase therapeutic results by bypassing traditional drug administration barriers and directing medication to the intended area. The ELISA results show that PVA/PVP microneedles loaded with KF-SP treat inflammatory and allergic eye problems better than KF solution or KF-SP. The microneedle system's enhanced efficacy in treating widespread ocular inflammation and allergy makes it a potential method for continuous, targeted medication administration in ophthalmology [9, 38]. The KF-SP-loaded microneedle system exhibited notable decreases in IgE, TNF-α, and IL-6 levels, highlighting its potent anti-inflammatory and immunomodulatory properties. Hypersensitivity reactions are marked by increased levels of IgE. The reduction indicates the system's capacity to modulate immune responses associated with allergic conjunctivitis. TNF-α and IL-6 are key pro-inflammatory cytokines that cause inflammation and tissue damage. Their suppression matches therapeutic efficacy, suggesting molecular inflammation reduction.Patients with allergic conjunctivitis experience less redness, swelling, and discomfort when these biomarkers decrease. These biomarkers are also important targets in corticosteroid and antihistamine treatments for allergic ocular conditions. These results match clinical responses, demonstrating the KF-SP system's translational potential. The current findings strongly suggest therapeutic relevance, but human clinical trials are needed to confirm these effects and their alignment with clinical outcomes.

## Histopathological examination results

**Fig. 11:**
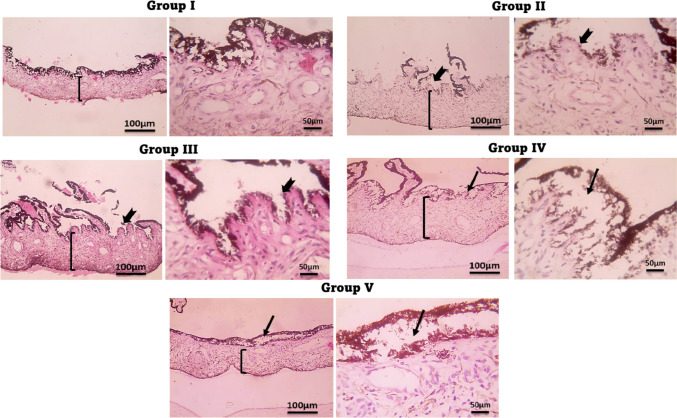
Microscopic histopathologic examination of the conjunctiva, in allergic conjunctivitis model rabbits after topical application of different Ketotifen fumarate formulations. **Group I**: normal group (normal group); **Group II**: Positive control (Diseased group); **Group III**: Treated by the KF suspension; **Group IV**: Treated by KF- SP; and **Group V**: Treated by KF-SP loaded PVA/PVP MNs patch H&E, 400 × . Notes: Edema (*) and dilated blood vessels (thin red arrows) in rabbits from histamine Stratified epithelia (arrows) were observed in the normal conjunctiva, while the normal submucosa displayed fibroblasts formed like stromal spindles (arrowheads). Strong inflammatory exudates primarily composed of macrophages, plasma cells (arrowheads), and eosinophilic scattering covering epithelia (arrows) were seen in the OVA/AH-treated group; these findings suggested hyperplasia and desquamation of the squamous epithelium

**Fig. 12:**
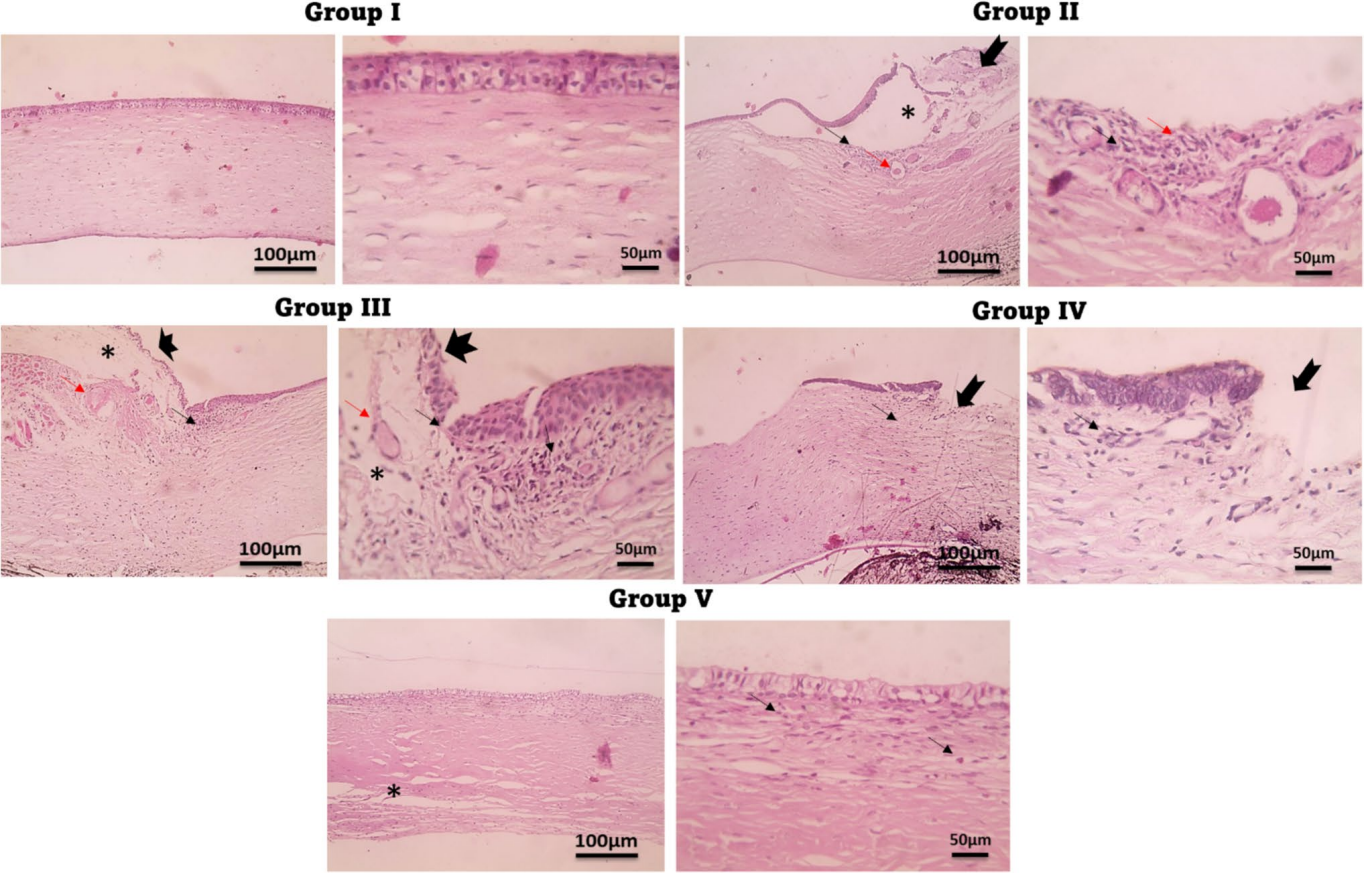
Microscopic histopathologic examination of the cornea in allergic conjunctivitis model rabbits after topical application of different Ketotifen fumarate formulations. **Group I**: normal group (normal group); **Group II**: Positive control (Diseased group); **Group III**: Treated by the KF suspension; **Group IV**: Treated by KF- SP; and **Group V**: Treated by KF-SP loaded PVA/PVP MNs patch, H&E, 400 × **Notes**: Edema (*) and dilated blood vessels (thin red arrows) in rabbits from histamine Stratified epithelia (arrows) were observed in the normal conjunctiva, while the normal submucosa displayed fibroblasts formed like stromal spindles (arrowheads). Strong inflammatory exudates primarily composed of macrophages, plasma cells (arrowheads), and eosinophilic scattering covering epithelia (arrows) were seen in the histamine-treated group; these findings suggested hyperplasia and desquamation of the squamous epithelium.

Figures 11 and 12 showed the histological analysis of the conjunctiva (A) and cornea (B) of rabbits respectively, exposed to different treatments yielded significant findings on the impact of (OVA/AH) -induced irritation and successive therapeutic interventions. The main focus of the study was to examine how irritated rabbit eyes react to various formulations, such as KF-SP systems, as well as KF-SP-loaded PVA/PVP microneedles (MNs).

In the normal control group **GI**, the conjunctiva and cornea showed normal histology, absent any indications of irritation or tissue injury. This group functioned as the reference point, verifying the anatomical integrity of the eye during typical circumstances. By contrast, the **GII** group (diseased group) showed significant damage induced by (OVA/AH) I.P injections. Administration of (OVA/AH) caused significant redness, swelling, ulceration, and infiltration of white blood cells in both the conjunctiva and cornea, therefore illustrating the prompt inflammatory response to (OVA/AH). The results indicate a notable decrease in ocular health within 30 min following the administration of (OVA/AH). The KF suspension group **G III** showed amelioration in ocular conditions in comparison to **G II**. Nevertheless, further histological analysis showed thicker conjunctiva, increased mucus production, diffuse vascular macula in the conjunctiva, corneal erosions, edema, and persistent leukocytic infiltration, despite the treatment. These findings indicate that whereas KF discontinuation offered some alleviation, it was inadequate in fully reversing the detrimental or inflammatory consequences induced by (OVA/AH). The **G IV** therapy group, which received KF-SP, had a more significant improvement marked by reduced swelling and partial regression of the normal conjunctival structure. Moreover, the cornea exhibited a reduced occurrence of inflammation, marked by a decrease in the infiltration of white blood cells and fewer formation of ulcers. The results indicate that the SP formulation of KF is more effective than the suspension version in minimizing the risk of retinal irritation [4]. The **GV** group, which received treatment with KF-SP-loaded PVA/PVP MNs, exhibited the most remarkable recovery, as indicated by the full restoration of the conjunctiva and cornea within 24 h. The histological examination revealed a significant reduction in edema, slight infiltration of white blood cells, and a recovery of the normal structural characteristics of the conjunctiva and cornea. The study showcases that the PVA/PVP microneedle system, including KF-SP, had superior efficacy and extended drug release, leading to rapid inflammation healing and tissue regeneration. This work demonstrates that several formulations of KF have diverse levels of effectiveness in reducing eye pain induced by (OVA/AH) in rabbit models. The findings of this work demonstrate that several formulations of KF exhibit diverse benefits in mitigating ocular pain induced by (OVA/AH) in rabbit model subjects. The conventional KF suspension (**G III**) partially decreased inflammation and tissue damage, while the SP formulation (**G IV**) further reduced them. The KF-SP-loaded PVA/PVP microneedles (**G V**) achieved the most rapid and thorough recovery of the conjunctiva and cornea. These findings demonstrate that the use of SP and microneedles can enhance the treatment of ocular inflammatory diseases by providing sustained medication delivery and minimizing eye irritation caused by (OVA/AH).

## Scalability and production challenges for KF-SP-loaded MNs

Scaling up production of KF-SP-loaded microneedles (MNs) presents a number of challenges in achieving industrial feasibility. The micromolding method is effective for research, but it requires modification for large-scale manufacturing to maintain size, shape, and drug loading. Variability in biocompatible polymers such as PVA and PVP, which are used to make MNs, can affect mechanical properties and dissolution profiles, necessitating consistency in material quality. Batch-to-batch consistency requires high-throughput equipment with precise control and integrated quality assurance. Due to MNs' dual classification as pharmaceuticals and medical devices, regulatory hurdles require extensive product quality, stability, and safety validation. Automated micromolding systems, material supply chain optimization, fabrication protocol standardization, and early regulatory engagement to streamline approvals are proposed solutions. These strategies aim to turn KF-SP-loaded MNs from a research innovation into a commercial product that meets clinical needs.

## Conclusion

This study reports on the efficacy of multiple strategies approach to a nano-spanlastic and dissolving microneedle (KF-SP-MN) system for treating allergic conjunctivitis. The PVA/PVP microneedle patch coated with KF-SP improves the bioavailability and therapeutic efficacy of ketotifen fumarate by regulating and prolonging drug release. In the enhanced formulation, levels of TGF-β and IL-10 increased, but levels of pro-inflammatory markers such as IgE, TNF-α, and IL-6 decreased. Research into gene expression has shown that modulating IGF-1, Annexin A1, and Bcl2 levels lend credence to their potential as medicines. Histological studies showed that the KF-SP-MN system sped up tissue repair compared to standard treatment by lowering inflammation in the eye and conjunctiva. By creating this new microneedle delivery method, the problems that came up when trying to give medicines to the eyes have been greatly reduced. Long-term use is okay as an AC treatment because it isn't painful and the healing benefits last a long time.

## Supplementary Information

Below is the link to the electronic supplementary material.

Supplementary file1 (RAR 157 KB)

## Data Availability

The authors confirm that the data supporting the findings of this study are available within the article.
